# Protein Extraction From FFPE Kidney Tissue Samples: A Review of the Literature and Characterization of Techniques

**DOI:** 10.3389/fmed.2021.657313

**Published:** 2021-05-13

**Authors:** Maria García-Vence, Maria del Pilar Chantada-Vazquez, Ana Sosa-Fajardo, Rebeca Agra, Ana Barcia de la Iglesia, Alfonso Otero-Glez, Miguel García-González, José M. Cameselle-Teijeiro, Cristina Nuñez, Juan J. Bravo, Susana B. Bravo

**Affiliations:** ^1^Proteomic Unit, Health Research Institute of Santiago de Compostela (IDIS), University Clinical Hospital of Santiago de Compostela (CHUS), Santiago de Compostela, Spain; ^2^Research Unit, Lucus Augusti University Hospital (HULA), Servizo Galego de Saúde (SERGAS), Lugo, Spain; ^3^Research Group of Industrial Microbiology and Food Biotechnology (IMDO), Vrije Universiteit, Brussels, Belgium; ^4^Nephrology Laboratory, Health Research Institute of Santiago de Compostela (IDIS), University Clinical Hospital of Santiago de Compostela (CHUS), Santiago de Compostela, Spain; ^5^Nephrology Service, University Clinical Hospital of Ourense (CHOU), Orense, Spain; ^6^Department of Pathology, Health Research Institute of Santiago de Compostela (IDIS), University Clinical Hospital of Santiago de Compostela (CHUS), Santiago de Compostela, Santiago, Spain; ^7^Nephrology Service, University Clinical Hospital of Vigo (Alvaro Cunqueiro-CHUVI), Vigo, Spain

**Keywords:** formalin-fixed paraffin-embedded, kidney tissue, deparaffinization, protein extraction, LC-MS/MS analysis

## Abstract

Most tissue biopsies from patients in hospital environments are formalin-fixed and paraffin-embedded (FFPE) for long-term storage. This fixation process produces a modification in the proteins called “crosslinks”, which improves protein stability necessary for their conservation. Currently, these samples are mainly used in clinical practice for performing immunohistochemical analysis, since these modifications do not suppose a drawback for this technique; however, crosslinks difficult the protein extraction process. Accordingly, these modifications make the development of a good protein extraction protocol necessary. Due to the specific characteristics of each tissue, the same extraction buffers or deparaffinization protocols are not equally effective in all cases. Therefore, it is necessary to obtain a specific protocol for each tissue. The present work aims to establish a deparaffinization and protein extraction protocol from FFPE kidney samples to obtain protein enough of high quality for the subsequent proteomic analysis. Different deparaffination, protocols and protein extraction buffers will be tested in FFPE kidney samples. The optimized conditions will be applied in the identification by LC-MS/MS analysis of proteins extracted from 5, 10, and 15 glomeruli obtained through the microdissection of FFPE renal samples.

## Introduction

Chronic diseases such as diabetes, hypertension, obesity, or chronic kidney disease have been studied for many years through physiological, biochemical, and genetic approaches, contributing to the understanding of the pathophysiology of these diseases (1–3). In the last decade, proteomics offered insights into the translation of biological information from DNA to RNA to proteins (4) and post-transcriptional modifications caused by different diseases.

**Figure 1:**
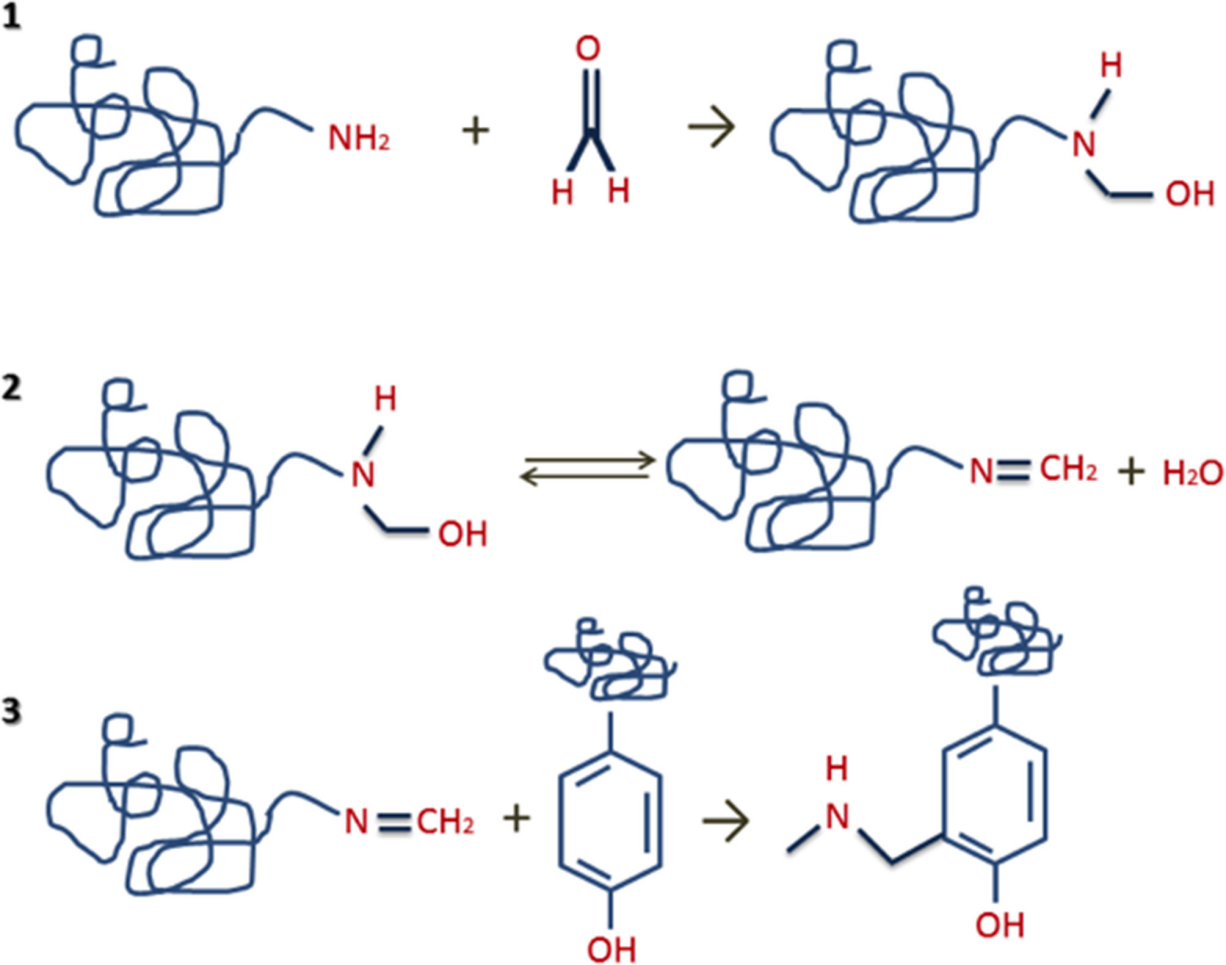
Formation of protein crosslinks steps: (1) reaction of formaldehyde with proteins/peptides to generate unstable methylol groups; (2) interaction of methylol groups with proteins/peptides to form Schiff bases through the elimination of water; (3) interaction of Schiff bases with proteins/peptides to form stable intra- or intermolecular “methylene bridges”.

In clinical practice, formalin-fixed and paraffin-embedded (FFPE) of tissue biopsies is a specific technique used to prepare and preserve tissue specimens utilized in research, examination, diagnostics, and drug development. (5–9). These samples represent an almost endless biorepository for DNA, RNA and protein analyses. However, one of the main problems is trying to extract proteins from FFPE specimens, due to the nature of the cross-links formed after the treatment with formalin (10–12) (see Figure 1). These molecular crosslinks difficult protein extraction and more aggressive extraction techniques than those used for fresh or frozen tissues are necessary (7, 11, 12).

However, in the last years, different proteomic analysis of FFPE specimens were reported (13). The first challenge found in the FFPE proteomics is the adaptation of heat-induced antigen retrieval methods, used for immunohistochemistry (IHC), to the extraction of the proteins from FFPE tissue to achieve proteomic approximations (7). In recent years, considerable advances have been made regarding the proteomic analysis of FFPE samples (9), improving the coverage and the number of proteins identified, studying post-translational modifications (9, 14), using iTRAQ-label-based proteomics or “label-free” assays for the quantitative analysis (8, 15). For example, recently, using a xylene-free method for the deparaffinization step and a combination of SDS or urea as buffers for the protein extraction step, were possible the identification and quantification of a set of proteins from FFPE tissues using a tandem mass tag (TMT) labeling approach (16).

In some cases, literature is confusing and contradictory (6, 17), being necessary standardization of some parameters as extraction buffers, pH, temperature, and pressure before the mass spectrometry analysis of proteins from FFPE specimens (9).

In relation to FFPE kidney tissue samples, the first study reported an enhancement method for immunohistochemical staining based on microwave oven heating of tissue sections in the presence of SDS-containing buffers (18). Two years later, one study developed with FFPE mouse kidney tissue samples shows good results after replacing the SDS extraction buffer with other buffers compatibles with the subsequent trypsin digestion and LC-MS/MS analysis (13). In 2009, a novel heat-induced antigen retrieval strategy using SDS-containing Laemmli buffer for efficient intact protein recovery from formalin-fixed tissues for subsequent analysis by western blotting were reported (19). A few years later, different proteins extraction buffers were tested in FFPE and frozen tissue samples. Among themselves, the RapiGest kit allows the identification of a high percentage of common proteins in both fresh and FFPE tissue samples (58% of the total of proteins identified) (20).

According to previous results (21), Perroud et al. (22) developed a method for the extraction of proteins from frozen and FFPE renal cell carcinoma (RCC) tissues samples which allowed the identification of 105 proteins significantly altered in RCC versus normal tissue. Sprung et al. (23) used the same method with minor modifications to test the reproducibility of multiple reaction monitoring (MRM) mass spectrometry-based peptide quantization in tryptic digests from frozen and FFPE clear cell renal cell carcinoma (ccRCC) tissues samples. A total of 1971 common proteins were identified in frozen and FFPE tissues samples showing that effect of formalin fixation in the proteins from tissues is not an obstacle for the development of the MRM mass spectrometry analysis (23).

An efficient and reproducible procedure for the extraction of proteins from FFPE renal tissues were also developed and optimized (24). This approach produces reliable results and biologically meaningful proteomic profiles generated by reverse-phase protein arrays RPPA, a high-throughput technology, which can detect changes in protein levels and protein functionality in numerous tissue and cell sources.

One year later, Craven et al. (15) developed a systematic analysis of normal and malignant FFPE renal tissues to examine the effect of paraffin-blocked samples' age/time storage and levels of technical and biological variability. The quantitative proteomic analysis revealed that the percentage of proteins common in normal and malignant tissues was remarkably high (around 60%).

In 2014, an study revealed that the combination of microdissection and tandem mass spectrometry could be used to investigate the proteome of isolated glomeruli from FFPE tissue in the non-clipped kidney of two-kidney one-clip (2K1C) hypertensive rats (25).

In 2015, stable isotopic dimethylation of primary amines was used for the first time for the quantitative proteomic analysis of FFPE clear cell renal cell carcinoma tissue samples without interference from formalin employed in the FFPE process (8). Proteome profiles changes in clear cell renal cell carcinoma (ccRCC) FFPE tissue specimens were compared with that of adjacent non-malignant renal tissue, finding differences in the levels of glycolytic enzymes, annexins as well as ribosomal and proteasomal proteins.

In the same year, Shen et al. (26) tested different protein extraction buffers as Zwittergent based buffer, SDS-containing buffer with/without polyethylene glycol 20000 (PEG20000), urea-containing buffer, and FFPE-FASP protein preparation kit in different types of rat FFPE tissues, including the heart, brain, liver, lung, and kidney. Their results show that Zwittergent buffer was the most efficient buffer for identifying peptides and proteins, compatible with mass spectrometry, in these different tissues.

And finally, in 2019, another work (27) shows again the use of SDS buffer as an effective method to obtain good identification by mass spectrometry in several tissues. An exhaustive revision about these methods in several different tissues was developed by Giusti et al. in the same year (28).

In summary, the method to obtain protein from FFPE samples is not well established and several major topics must be addressed to assure the scientific accuracy of FFPE tissue proteomics. Moreover, the selection of the deparaffinization method and the protein extraction buffer depends on the tissue analyzed. Therefore, it is important to consider that these methods may vary based on the procedure purpose and they also present some limitations, as the difficulty to identify low abundant proteins and post-translational modifications (PTMs) (29).

The principal aim of the present work is to establish a new extraction protocol for obtaining non-degraded proteins from FFPE kidney biopsy specimens and to test the minimum amount of tissue needed to obtain enough protein with good quality for the subsequent mass spectrometry (LC-MS/MS) analysis. These optimized conditions will be tested in microdissected glomeruli for the future discovery of biomarkers in diseases as glomerulonephritis.

## Materials and Methods

### Chemicals

All reagents used were HPLC grade or higher. Trizma base, glycerol, Triton X-100, CHAPS, tributyl phosphine, sodium deoxycholate, α-cyano-4-hydroxy-cinnamic acid, EDTA, sodium chloride (NaCl), urea, thiourea, trifluoracetic acid (TFA), ammonium persulfate (APS) and iodoacetamide (IAA) were purchased from Sigma-Aldrich (United States). Dithiothreitol (DTT), Coomassie Blue and acrylamide/bis-acrylamide was purchased from Serva (Germany). Trypsin sequence-grade was purchased from Promega (United States). Sodium dodecyl sulfate (SDS), *N,N,N',N'*-tetramethylethylene-diamine (TEMED) and a molecular scale marker for gel electrophoresis were purchased from Bio-Rad (United States). Formic acid (HCOOH), ammonium bicarbonate (Ambic), methanol (MeOH), ethanol (EtOH), acetonitrile (ACN), isobutanol, hydrochloric acid (HCl), glacial acetic acid and xylene were purchased from Scharlau (Spain).

### Biological Samples

All samples were obtained through collaboration with Dr. Miguel Garcia from the Nephrology Laboratory (NefroChus), Health Research Institute of Santiago de Compostela (IDIS). Initial standardization will be developed with sections of FFPE kidney samples from healthy patients. These sections [one section of 3 μm (over 3.5 mg) or 1 μm (over 1.5 mg)] were cut in 3 mm pieces to facilitate the deparaffinization process, therefore, 4–5 mg of sample per tube we used in the first approach (3 μm), and a decreasing amount 1.5 mg (1 μm) was used in the second approach to pinpoint the sensibility of the process.

The experiment was conducted in conformity with the declaration of Helsinki and approved by the Clinical Research Ethics Committees (CEIC) of Galicia (Spain) with approval number 2017/357.

### Deparaffinization Methods

Three deparaffinization methods reported in the literature were followed (see Table 1):

- **First method**: FFPE samples were incubated (x*3*) with 1 mL of xylene for 5 min at 65°C under vortex, instead of washing them. The rehydration of the sample was then performed by incubation with decreasing alcohol percentages (100, 85, and 75% EtOH) for 5 min at room temperature (RT). After that, samples were allowed to air dry before the extraction step (15).
- **Second method**: FFPE samples were incubated (x*3*) with 150 μL of xylene for 5 min at RT. The rehydration of the sample was then performed by incubation with decreasing alcohol percentages (100, 90, 80, and 70% EtOH) for 1 min at RT. Finally, samples were left in milli-Q H_2_O for 30 min (24, 26, 30).
- **Third method**: FFPE samples were incubated with 600 μL of mineral oil for 2 min at 95°C, and then centrifuged at 14,000*g* for 1 min. After that 350 μL of mineral oil was added for 5 min at 95°C. Mineral oil was then removed before the protein extraction step (31).

**Table 1:** Deparaffinization methods reported in the literature that were followed in the present work. While in the deparaffinization with xylene (at 65°C and RT) is necessary a subsequent rehydration step with ethanol (in different percentages), the deparafinization with mineral oil (at 95°C) does not require this rehydration step.

	**Lysis Buffer Name**	**Composition**	**References**
1	Tris-SDS LB	100 mM Tris-HCl pH 8, 100 mM DTT, 4% SDS	(14, 25, 26)
2	Urea LB	5 M urea, 2 M thiourea, 2 mM tributylphosphine, 65 mM DTT, 4% CHAPS	(14, 26, 31)
3	Tris-SDS-DTT-Glycerol LB	62.5 mM Tris-HCl pH 6.8, 4% w/v SDS, 10% v/v glycerol, 100 mM DTT	(15)
4	Citrate-SDS LB	200 mM Tris-HCl, pH 7.5, 200 mM NaCl, 5% SDS and 100 mM sodium citrate	(31, 32)
5	SDS-LB	200 mM Tris-HCl pH 6.8, 2% w/v SDS, 20% v/v glycerol	(31, 32)
6	RIPA LB	150 mM NaCl, 10 mM Tris-HCl, pH 7.2, 2% SDS, 1% Triton X-100, 1% sodium deoxycholate, 5 mM EDTA	(22)

After the deparaffinization step, samples were frozen overnight before the protein extraction process.

### Protein Extraction

After the deparaffinization of the samples, proteins from FFPE kidney tissues specimens were extracted testing three different protocols for the disaggregation of the samples:

- **First protocol**: 50 μL of buffer were added, then the tissue was pressed through a 20G syringe and incubated in ice for 20 min, centrifuged and the supernatant collected (32).
- **Second protocol**: 50 μL of buffer were added to the sample, then boiled at 100°C for 15 min and centrifuged at 14,000*g* for 15 min at 4°C (31).
- **Third protocol:** 50 μL of buffer were added to the sample, then boiled for 20 min at 100°C plus 2 h at 60°C and then sonicated for 30 min (15). In the present work, the sonication step was substituted by a disaggregation step with a TissueLyser (Qiagen) (that disrupts tissues through high-speed shaking) for 3 min to improve tissue rupture, and then centrifuged at 14,000 rpm for 20 min to remove tissue remnants.

The composition of the different buffers used for the protein extraction step is described below (see Table 2):

- **buffer 1** (Tris-SDS LB): 100 mM tris pH 8, 100 mM DTT, 4% SDS (14, 25, 26).
- **buffer 2** (urea LB): 5 M urea, 2 M thiourea, 2 mM tributylphosphine, 65 mM DTT, 4% CHAPS (14, 26, 31).
- **buffer 3** (Tris-SDS-DTT-Gly LB): 62.5 mM Tris-HCl pH 6.8, 4% w/v SDS, 10% v/v glycerol, 100 mM DTT (15).
- **buffer 4** (citrate-SDS LB): 200 mM Tris-HCl, pH 7.5, 200 mM NaCl, 5% SDS 100 mM sodium citrate (31, 32).
- **buffer 5** (SDS-LB): 200 mM Tris-HCl pH 6.8, 2% w/v SDS, 20% v/v glycerol (31, 32).
- **buffer 6** (RIPA LB): 150 mm NaCl, 10 mm Tris-HCl, pH 7.2, 2% SDS, 1% Triton X-100, 1% sodium deoxycholate, 5 mm EDTA (22).

**Table 2:** Composition of the different protein extraction buffers reported in the literature that were used in the present work.

**Deparaffinization**	**Rehydration**	**References**
Xylene - 65°C	ETOH 100% ETOH 85% ETOH 70%	(15)
Xylene - RT	ETOH 100% ETOH 90% ETOH 80% ETOH 70%	(14, 24, 26)
Mineral oil 95°C2 min		(31)

Protein quantification was developed with an RCDC kit (BioRad, USA) following the manufacturer's instructions. Each different condition was tested in three different FFPE kidney tissue samples (*x3*).

### One Dimensional SDS-PAGE

One dimensional sodium dodecyl sulfate polyacrylamide gel electrophoresis (1D SDS-PAGE) was performed to test the protein quality and degradation. 20 μL of each extraction were loaded in a 10% acrylamide/bis-acrylamide gel and run-in a Plus Dodeca electrophoresis cell (BioRad, USA) at constant amperage of 30 mA per gel for 4 h, until the front line reached the lower edge. The gels were stained with Coomassie Blue for 2 h at room temperature under agitation and distained with MeOH/acetic acid (45/7.5%) for 12 h, also under agitation. They were then washed in milli-Q H_2_O and scanned.

A study of the global proteome was also done using acrylamide/bis-acrylamide gel leaving only a 3 mm space in the gel separating phase (resolving phase) then stopping the electrophoresis (33, 34). The gels were stained with Coomassie Blue for 2 h at room temperature under agitation and distained with MeOH/acetic acid (45/7.5%) for 12 h. They were then washed in milli-Q H_2_O and scanned. The obtained bands were cut and processed through “in-gel” digestion.

### “In-gel” Protein Digestion

The “in-gel” protein digestion was manually performed according to the Shevchenko et al. protocol (35) with minor modifications. Briefly, the selected bands from 1-DE gels were excised and washed with a solution containing 50 mM Ambic and 50% MeOH. Proteins were reduced with 10 mM DTT in 50 mM Ambic and alkylated with 55 mM IAA in 50 mM Ambic, and subsequently rinsed with 50 mM Ambic in 50% MeOH, dehydrated through the addition of ACN and dried in a SpeedVac (Thermo Scientific, USA). Modified porcine trypsin was added to the dried gel slices at a final concentration of 20 ng/μL in 20 mM Ambic, followed by incubation at 37 °C for 16 h. The peptides were extracted three times by incubation in 40 μL of 60% ACN in 0.5% HCOOH for 20 min. The resulting peptide extracts were pooled, concentrated, and dried in a SpeedVac and stored at −20°C until their analysis through LC-MS/MS.

### Protein Identification by Mass Spectrometry (LC-MS/MS) and Data Analysis

The analysis by LC-MS/MS was performed as described previously by our group and different authors (36–40). Briefly, a TripleTOF 6600 System (SCIEX, Foster City, CA) was employed for the Data acquisition using a Data-dependent workflow. After MS/MS analysis, data files were processed using ProteinPilot^TM^ 5.0.1 software from SCIEX which uses the algorithm Paragon^TM^ for database search and Progroup^TM^ for data grouping. Data were searched using a Human specific Uniprot database. False discovery rate was performed using a non-lineal fitting method displaying only those results that reported a 1% Global false discovery rate or better. Each determination was performed in triplicate (*x3*).

Protein ontology classification was analyzed using the tool PANTHER (http://www.pantherdb.org/). The differentially expressed proteins were grouped according to their molecular function, biological process, and cellular component.

### Laser Capture Microdissection of FFPE Glomeruli

Laser capture microdissection was performed using the laser microdissection system LEICA **AS LMD** (Leica Microsystems, Wetzlar, Germany) (41) containing a VSL-337ND-s Nitrogen Laser. FFPE renal samples were used to obtain 5, 10, and 15 glomeruli. These glomeruli were laser microdissected from 3 μm sections and pressure catapulted into a tube cap (AdhesiveCap 500 clear, Zeiss). Glomeruli with severe morphological damage were excluded as the objective was to investigate the minimal amount of tissue to obtain good quality protein. The total volume of dissected glomerular tissue ranged from 7.8 to 12.5 μL. Dissected FFPE tissue was stored at −20°C until deparaffinization. All analysis were performed in triplicate.

## Results

### Protein Yield and Quality of the Extraction Method

In the present work, FFPE kidney tissues samples (*x*3) were deparaffinated using xylene or mineral oil (see section **Deparaffinization Methods**), and the protein extraction from these samples were performed with three disaggregation protocols and six different extraction buffers (see section **Protein Extraction**).

**Figure 2:**
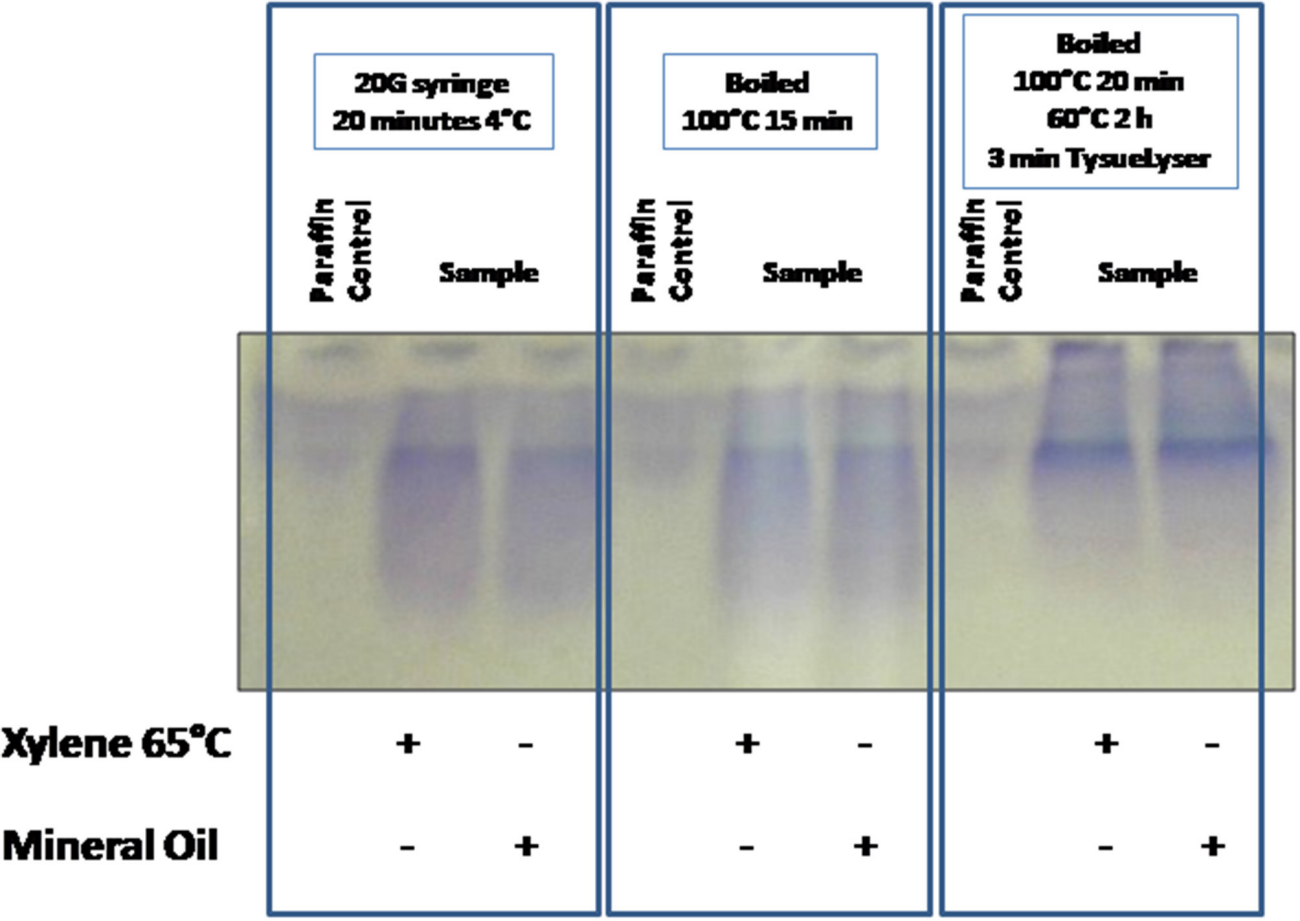
Comparison of the tissue disaggregation methods (protocols 1–3). 1D SDS-PAGE 10% gels were performed to evaluate the protein integrity. Deparaffinization with xylene at 65°C or with mineral oil at 95°C, and protein extraction **buffer 6** (RIPA LB) were selected.

Firstly, one experiment was developed to evaluate the protein integrity through 1D SDS-PAGE after the application of the three protein extraction protocols for the disaggregation of the samples. FFPE kidney tissue samples were deparaffinated with xylene at 65°C or mineral oil at 95°C, and the buffer selected for the extraction step was **buffer 6** (RIPA LB). Figure 2 compares the results obtained using the different extraction protocols. Visible bands were observed in the 1D SDS-PAGE gels with the three protocols; however, only well-defined bands were observed after using the **protocol 3** (see section **Protein Extraction**) with both deparaffinization solvents (paraffin was used as negative control). Probably, the high efficiency of the **protocol 3** is due to the combination of high incubation temperature with the TissueLyser for the disaggregation of the sample.

**Figure 3:**
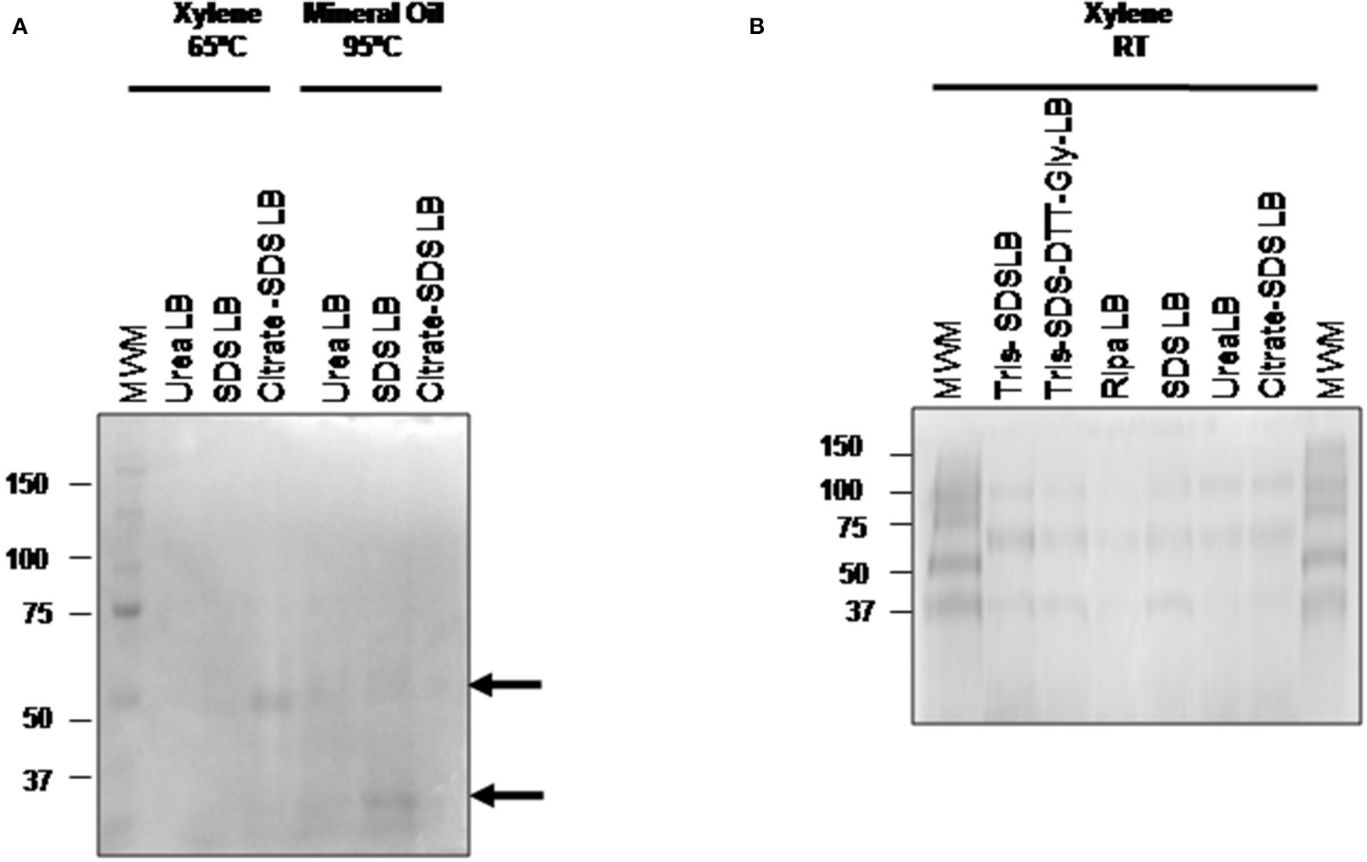
Comparison of the three different deparafinization methods and the protein extraction buffers efficiency. 1D SDS-PAGE 10% gels were performed to evaluate the protein integrity. **(A)** Visualization of gel protein bands after the deparaffinization with xylene at 65°C or with mineral oil at 95°C using the protein extraction buffers: **buffer 2** (urea LB), **buffer 4** (citrate SDS LB), and **buffer 5** (SDS LB). **(B)** Visualization of gel protein bands after the deparaffinization with xylene at RT using the protein extraction buffers: **buffer 1** (TRIS SDS LB), **buffer 2** (urea LB), **buffer 3** (TRIS SDS DTT GLY LB), **buffer 4** (citrate SDS-LB), **buffer 5** (SDS LB), and **buffer 6** (Ripa LB). All samples were treated under the conditions of protocol 3 (disaggregation with the TissueLyser).

After the optimization and selection of the best tissue disaggregation method (**protocol 3**), the deparaffinization efficiency of xylene (a toxic polar solvent) and mineral oil was compared using three different protein extraction buffers: **buffer 2** (urea LB), **buffer 4** (citrate-SDS LB) and **buffer 5** (SDS LB) (see Figure 3A). Better results were observed after the combination of the deparaffinization with xylene at 65°C and the **buffer 4**, and the deparaffinization with mineral oil at 95°C and **buffer 2** or **buffer 5**. To improve the deparaffinization with xylene, we tested the effect of temperature in this process following a method described previously by Araújo et al. (42). As Figure 3B shows, the deparaffinization of the FFPE tissues samples with xylene at RT in combination with all extraction buffers (**buffer 1 to 6**) and **protocol 3** (disaggregation with the TissueLyser) improved the protein extraction showing visible bands in all cases.

**Figure 4:**
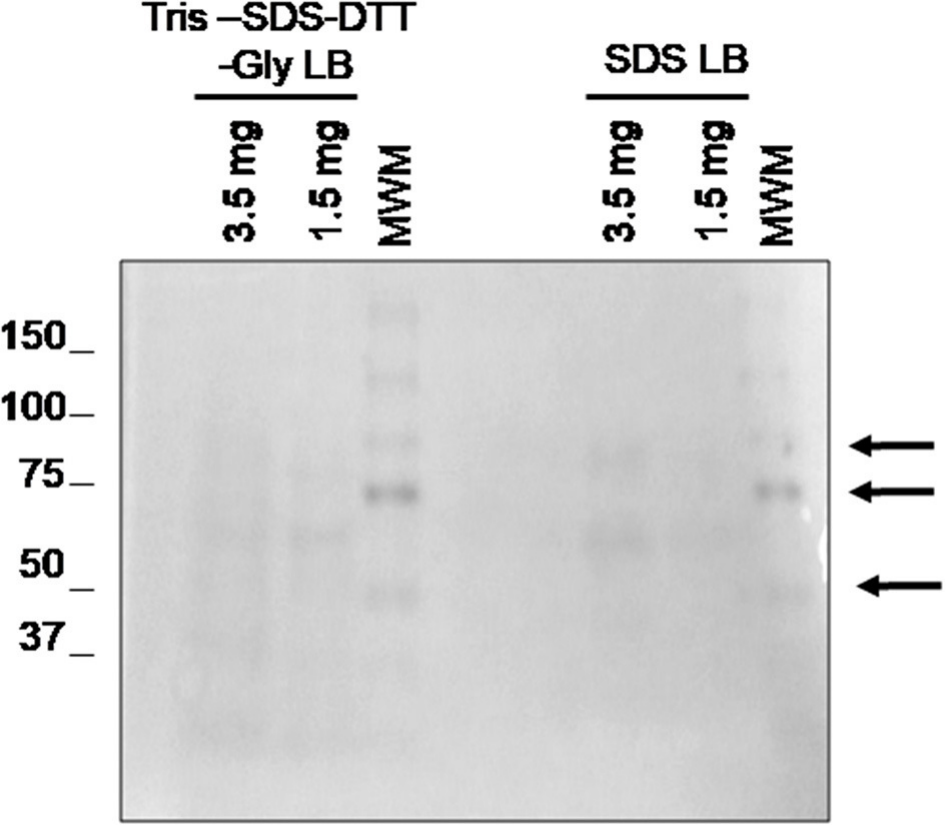
Evaluation of the minimal sample amount necessary to obtain good quality protein using 1.5 and 3.5 mg of tissue. 1D SDS-PAGE 10% gel was performed to evaluate the protein integrity. Visualization of gel protein bands after the deparaffinization of with xylene at RT using two protein extraction SDS-containing buffers: **buffer 3** (TRIS SDS DTT GLY LB) and **buffer 5** (SDS LB). All samples were treated under the conditions of protocol 3 (disaggregation with the TissueLyser).

As we mentioned above, this work also aims to evaluate the minimum amount of tissue needed to obtain enough protein with good quality for the subsequent mass spectrometry (LC-MS/MS) analysis. The previous optimization was made using 1 section of 3 μm of FFPE sample (3.5 mg of tissue), which is less than sections of 10 μm used by other authors. To assess if the procedure was still applicable to smaller quantities of FFPE samples, the optimized protocol was applied in one section of 3 μm (3.5 mg of tissue) and one section of 1 μm (1.5 mg of tissue). Surprisingly, Figure 4 shows that even using 1.5 mg of tissue, good quality proteins could be extracted using two SDS-containing buffers: **buffer 3** (Tris-SDS-DTT-Glycerol LB) and **buffer 5** (SDS LB).

### Mass Spectrometry (LC-MS/MS) Analysis for Protein Identification

Previous optimization showed that the best conditions for the treatment of FFPE kidney tissues samples consist of the deparaffinization with xylene at RT (see section **Deparaffinization Methods**), and a disaggregation step of the samples with a TissueLyser (**protocol 3**). After that, the efficiency of the six protein extraction buffers was evaluated by *LC-MS/MS* using a Triple TOF technology, performing each determination in triplicate (*x3*).

**Figure 5:**
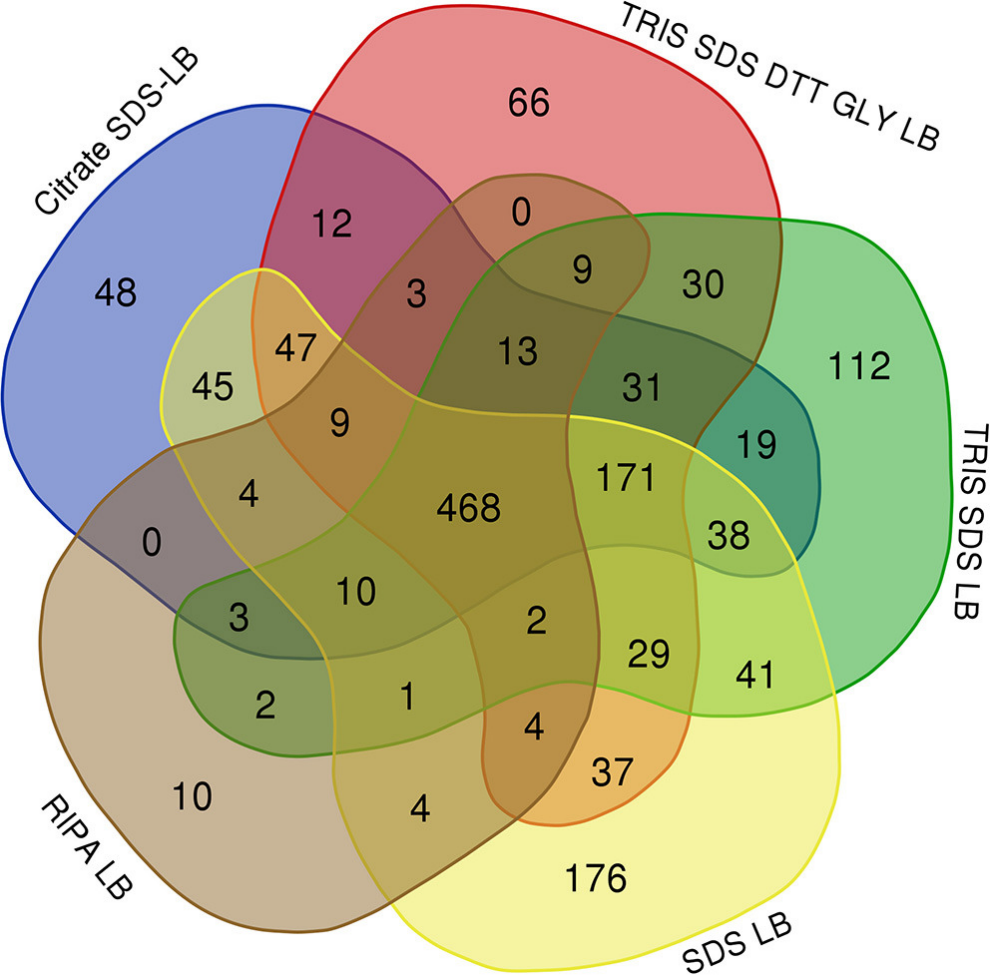
Venn diagram showing the number of common and unique proteins identified after using the different SDS-containing buffers for the extraction of proteins from FFPE kidney tissue samples: **buffer 1** (Tris-SDS LB), **buffer 3** (Tris-SDS-DTT-Glycerol LB), **buffer 4** (citrate-SDS LB), **buffer 5** (SDS-LB), and **buffer 6** (RIPA LB).

Table 3 shows the number of proteins identified after the use of the different protein extraction buffers (see Supplementary Table 1), being higher with SDS-containing buffers. While 468 proteins were commonly identified using the five SDS-containing buffers, a total of 112, 66, 48, 176, and 10 were unique proteins for the **buffer 1** (Tris-SDS LB), **buffer 3** (Tris-SDS-DTT-Glycerol LB), **buffer 4** (citrate SDS LB), **buffer 5** (SDS LB) and **buffer 6** (RIPA LB), respectively (see Figure 5 and Supplementary Table 2).

**Table 3:** Number of identified proteins by LC-MS/MS using the six different extraction buffers. There are shown the number of common proteins identified in all replicates (×3).

	**Buffer**	**number of Identified proteins**
1	TRIS SDS LB	979
2	Urea LB	569
3	TRIS SDS DTT GLY LB	930
4	Citrate SDS-LB	921
5	SDS LB	1,086
6	Ripa LB	540

**Figure 6:**
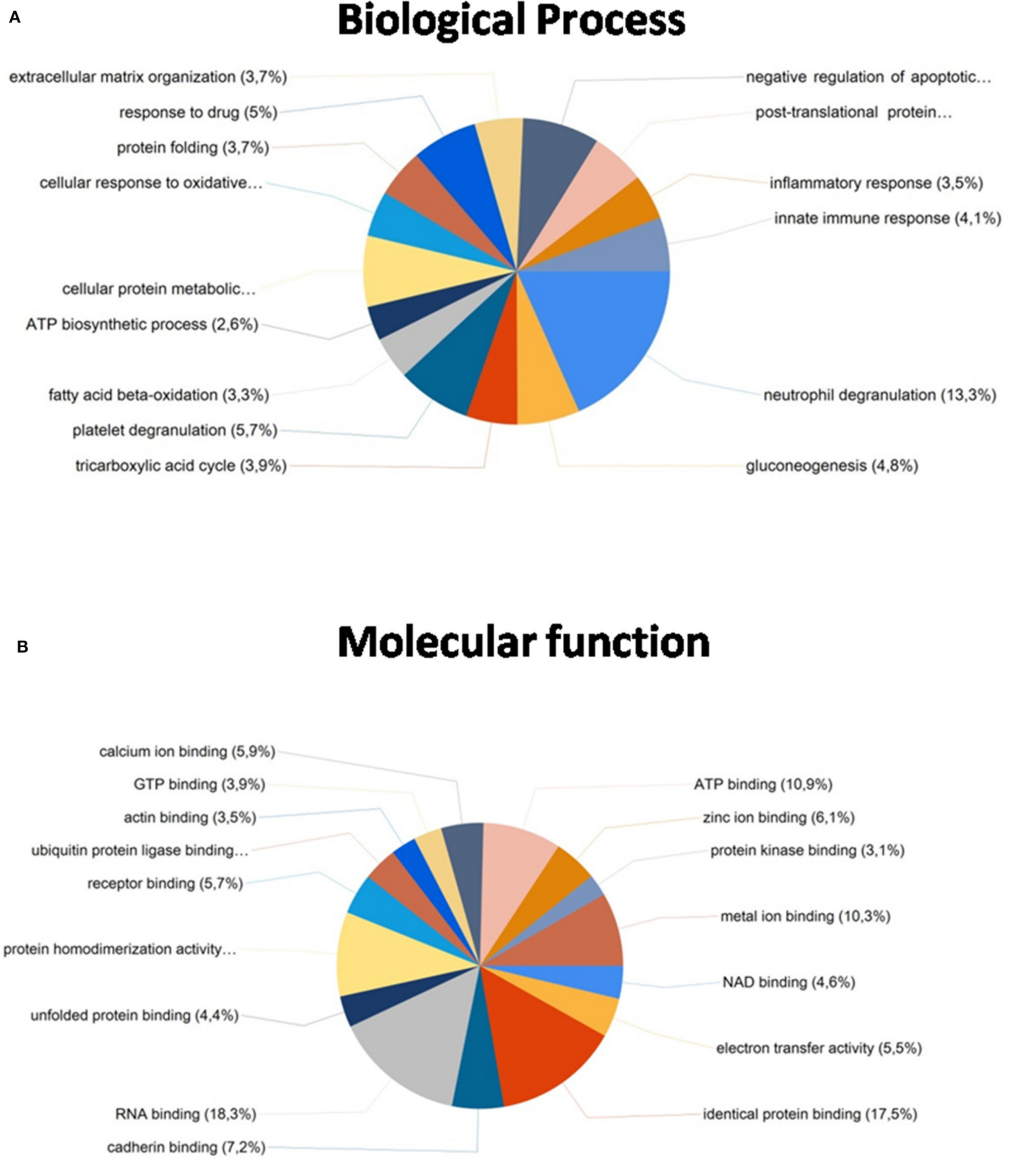
Functional classification of the 468 common proteins identified after the extraction with the five SDS-containing buffers according to their **(A)** biological process and **(B)** molecular function, using the FunRich program.

Importantly, from these common proteins, more than 80% were found to be mainly expressed in kidney tissue in databases as Uniprot (https://www.uniprot.org/) and were also identified in previously reported articles with FFPE kidney samples (25, 26, 42, 43). A functional analysis of these 468 common proteins were developed using the software FunRich (*http://www.funrich.org/*). Proteins were classified according to different criteria as their biological process (see Figure 6A) and molecular function (see Figure 6B) (see Supplementary Table 3). As Figure 6A shows, proteins identified were involved in the extracellular matrix organization (3.7%), protein folding (5%), inflammatory response (3.5%), innate immune response (4.1%) or ATP biosynthesis process (2.6%). In relation with their molecular function (see Figure 6B), most of the identified proteins correspond to ATP binding (10.9%), identical protein binding (17.5%), actin-binding (3.5%), receptor binding (5.7%) or RNA binding (18.3%).

**Figure 7:**
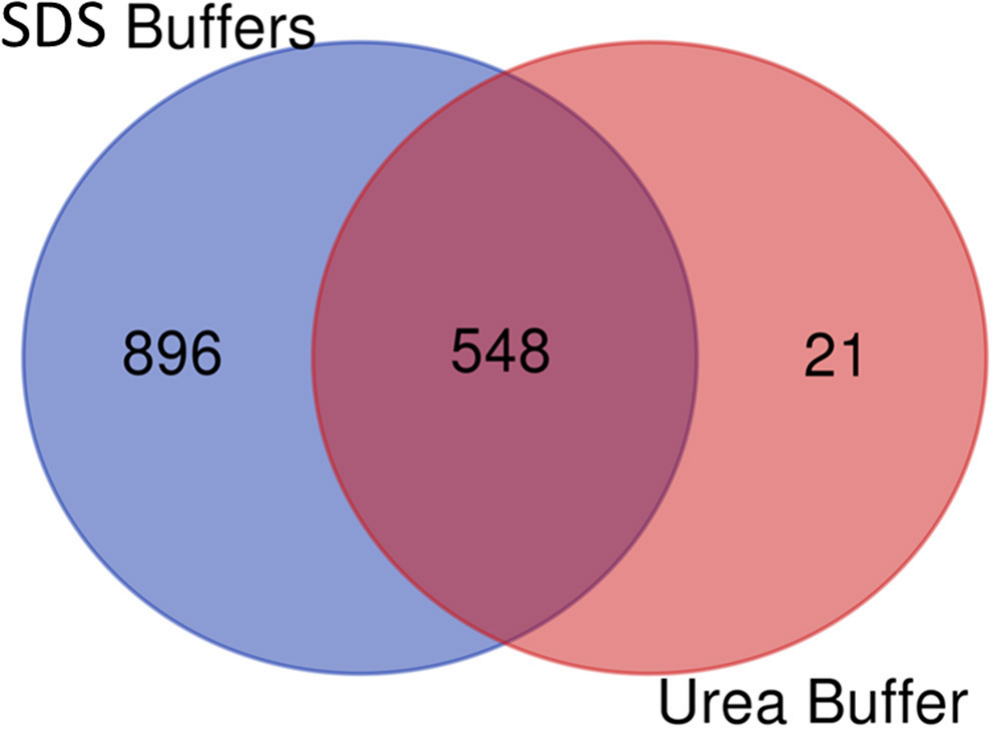
Venn diagram showing the number of common and unique proteins identified after using SDS-containing buffers [**buffer 1** (Tris-SDS LB), **buffer 3** (Tris-SDS-DTT-Glycerol LB), **buffer 4** (citrate-SDS LB), **buffer 5** (SDS-LB), and **buffer 6** (RIPA LB)] and the urea-containing buffer [**buffer 2** (urea LB)].

**Figure 8:**
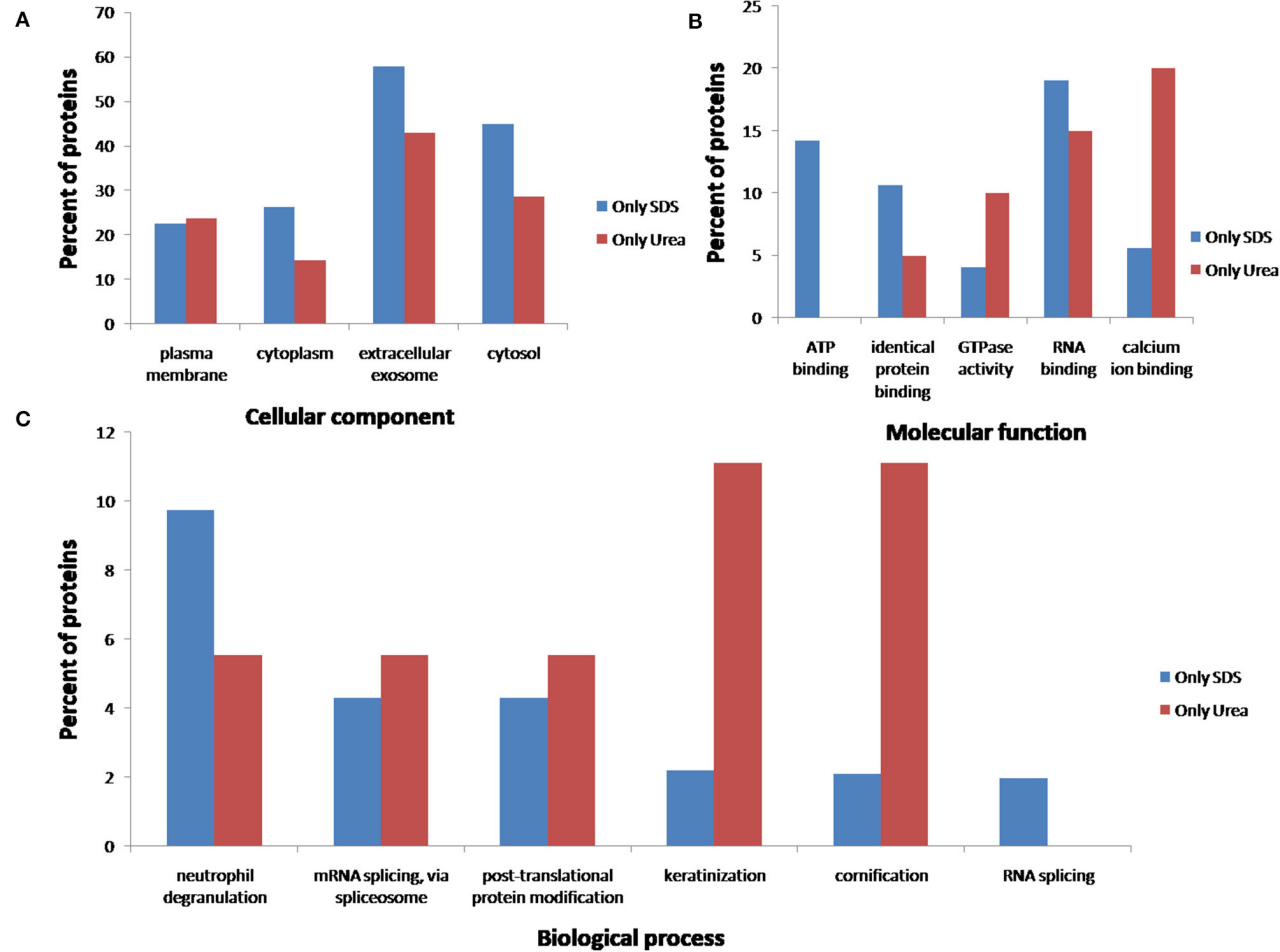
Functional classification of the 896 and 21 unique proteins identified after the extraction with the five SDS-containing buffers [**buffer 1** (Tris-SDS LB), **buffer 3** (Tris-SDS-DTT-Glycerol LB), **buffer 4** (citrate-SDS LB), **buffer 5** (SDS-LB), and **buffer 6** (RIPA LB)] (blue bars) and the urea-containing buffer [**buffer 2** (urea LB)] (red bars), respectively, according to their **(A)** cellular component, **(B)** molecular function, and **(B)** biological process, using the FunRich program.

After comparing total proteins (common and non-common) identified after the use of the five SDS-containing buffers (**buffer 1**, **buffer 3**, **buffer 4**, **buffer 5**, **buffer 6**) with the total of proteins identified after the use of the urea-containing extraction buffer (**buffer 2**: urea LB) (see Figure 7 and Supplementary Table 4), 548 proteins were common, 896 were unique proteins of the SDS-containing buffers and 21 uniques proteins of the urea-containing buffer. Figure 8 compares the functional analysis of the cellular component, biological process and molecular function of the unique proteins identified after the use of SDS-containing buffers and the urea-containing buffer (see Supplementary Table 4). As it could be observed, the different buffer composition affects the kind of proteins extracted from the FFPE kidney tissue specimens. In this sense, while with the SDS-containing buffers proteins identified were mainly located in the cytoplasm, cytosol, and extracellular exosomes, with the urea-containing buffer proteins identified were in the membrane (see Figure 8A). In relation with the molecular function, proteins identified with the SDS-containing buffers participates mainly in protein binding, RNA binding, or ATP binding function, proteins identified with the use of the urea-containing buffer are implicated in calcium-binding and GTPase activity (see Figure 8B). Finally, when analyzing the biological processes in both conditions, higher proteins were identified related to neutrophil degranulation and RNA splicing using SDS-containing buffers, and more proteins are related to mRNA splicing via spliceosome, post-translational protein modification, keratinization, and cornification after the use of the urea-containing buffer (see Figure 8C).

**Figure 9:**
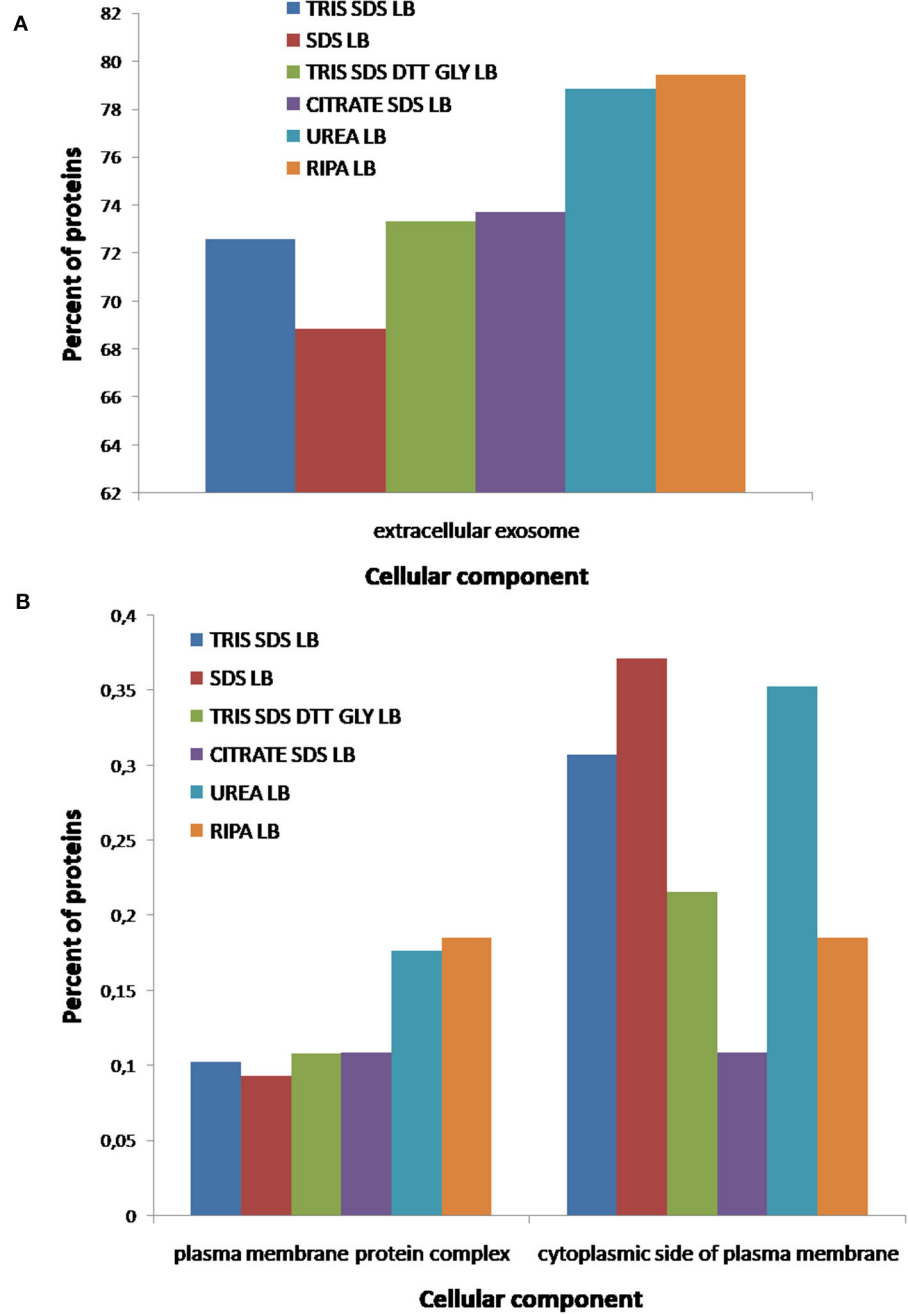
Functional classification of all proteins identified after the extraction with the six different buffers [**buffer 1** (Tris-SDS LB), *n* = 979 proteins; **buffer 2** (urea LB), *n* = 569 proteins; **buffer 3** (Tris-SDS-DTT-Glycerol LB), *n* = 930 proteins; **buffer 4** (citrate-SDS LB), *n* = 921 proteins; **buffer 5** (SDS-LB), *n* = 1,086 proteins; **buffer 6** (RIPA LB), *n* = 540 proteins], according to their cellular component: **(A)** extracellular exosomes, **(B)** plasma membrane complex and cytoplasmatic side of plasma membrane, using the FunRich program.

**Figure 10:**
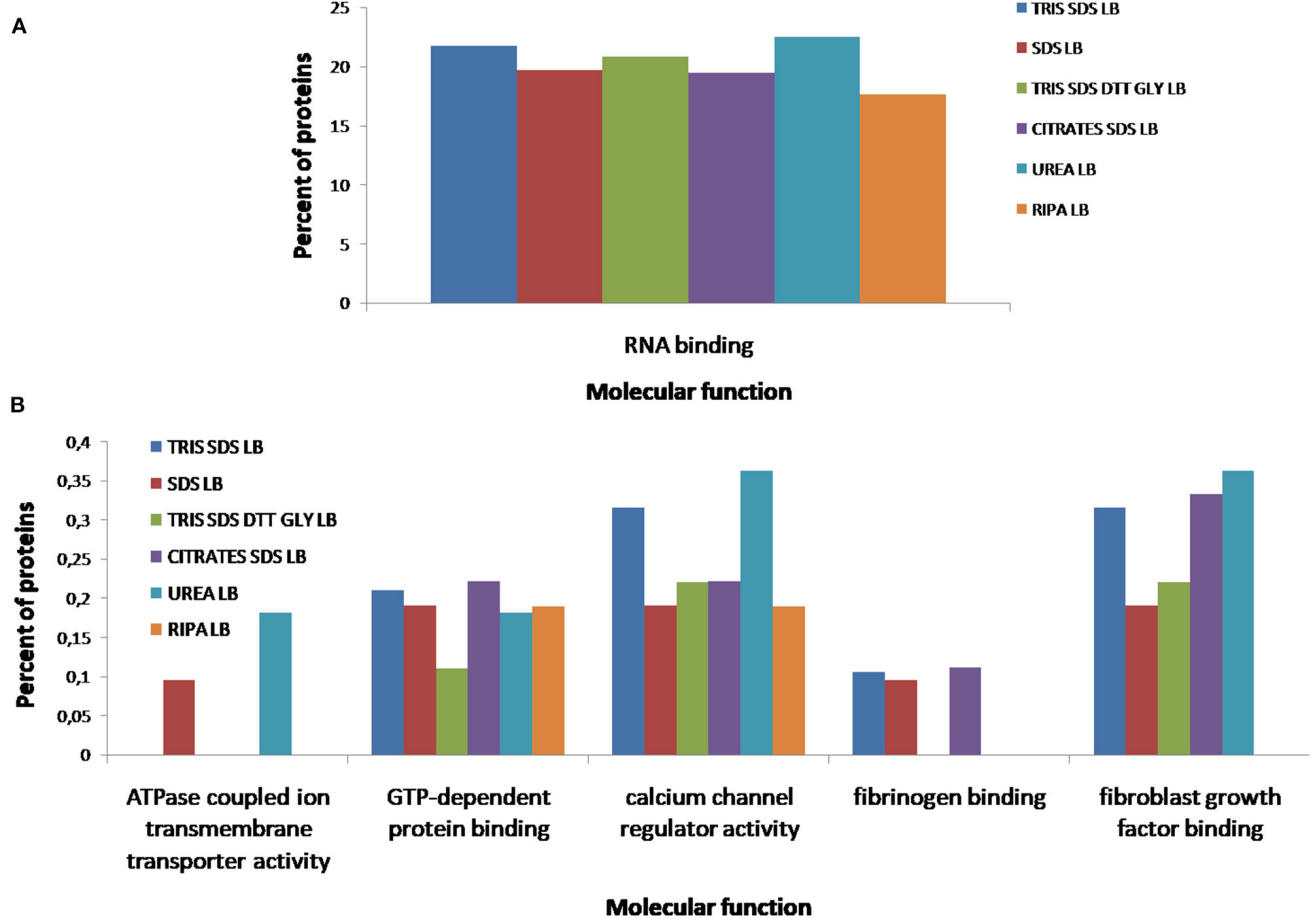
Functional classification of all proteins identified after the extraction with the six different buffers [**buffer 1** (Tris-SDS LB), *n* = 979 proteins; **buffer 2** (urea LB), *n* = 569 proteins; **buffer 3** (Tris-SDS-DTT-Glycerol LB), *n* = 930 proteins; **buffer 4** (citrate-SDS LB), *n* = 921 proteins; **buffer 5** (SDS-LB), *n* = 1,086 proteins; **buffer 6** (RIPA LB), *n* = 540 proteins], according to their molecular function: **(A)** RNA binding, **(B)** ATPase coupled ion transmembrane transporter activity, GTP-dependent protein binding, calcium channel regulator activity, fibrinogen binding, fibroblast growth factor binding, using the FunRich program.

**Figure 11:**
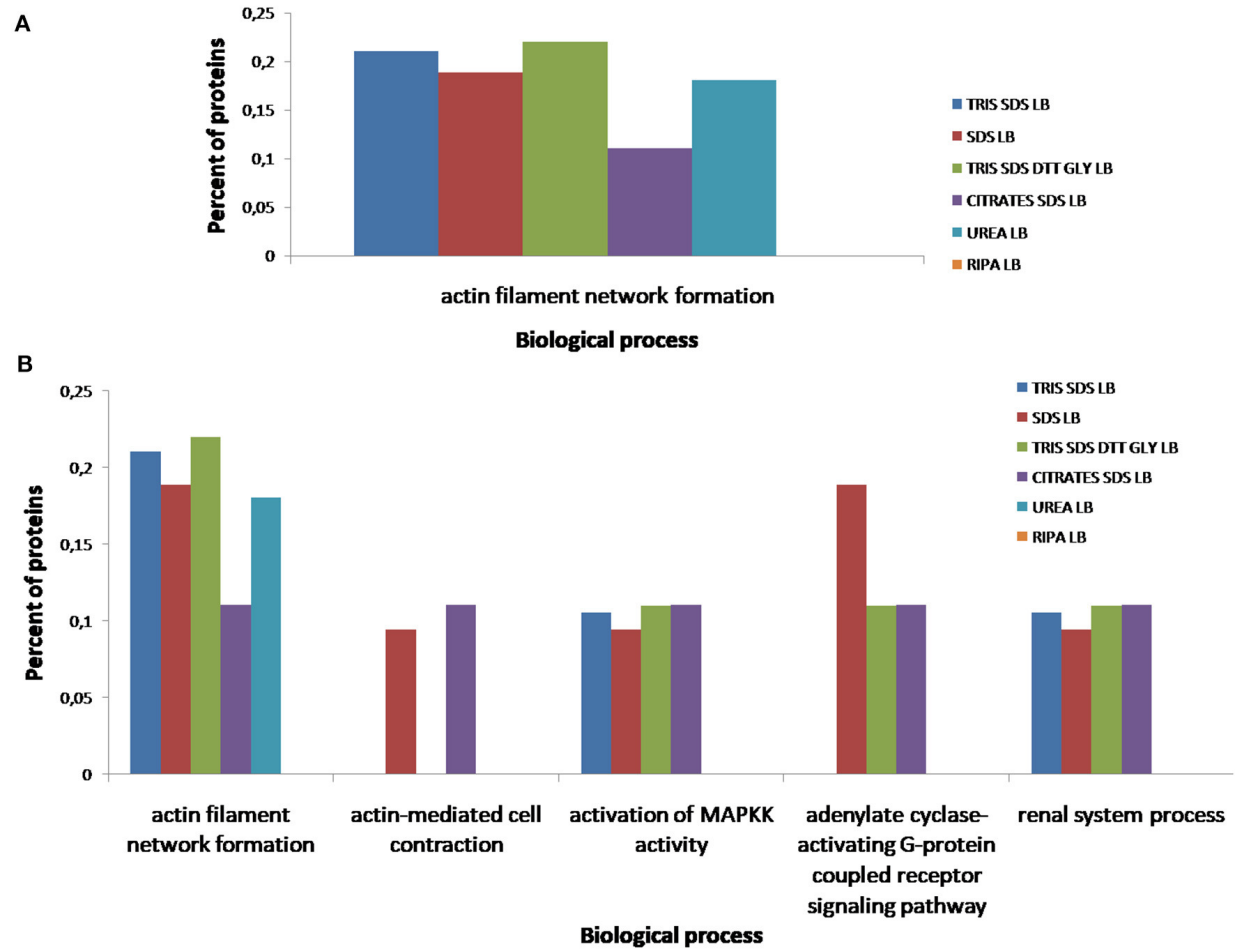
Functional classification of all proteins identified after the extraction with the six different buffers [**buffer 1** (Tris-SDS LB), *n* = 979 proteins; **buffer 2** (urea LB), *n* = 569 proteins; **buffer 3** (Tris-SDS-DTT-Glycerol LB), *n* = 930 proteins; **buffer 4** (citrate-SDS LB), *n* = 921 proteins; **buffer 5** (SDS-LB), *n* = 1,086 proteins; **buffer 6** (RIPA LB), *n* = 540 proteins], according to their biological process: **(A)** actin filament network formation, **(B)** actin-mediated cell contraction, activation of MAPKK activity, adenylate cyclase-activating G-protein coupled receptor signaling pathway, renal system process, using the FunRich program.

Finally, individual functional analysis of the proteins identified using each buffer was performed to evaluate the influence of the buffer composition on the extraction of a proteins with different properties/functionalities (see Figures 9–**11**, and Supplementary Table 5). Figure 9A shows that the number of proteins extracted from extracellular exosomes with **buffer 1** (Tris-SDS LB), **buffer 3** (Tris-SDS-DTT-glycerol LB) and **buffer 4** (citrate-SDS LB) were similar (between 72 and 74%), with **buffer 5** (SDS LB) is 68% and with **buffer 2** (urea LB) and **buffer 6** (RIPA LB) are 78–80% (see Figure 9A). Other cellular components, such as the plasma membrane protein complex, show the same profile (see Figure 9B). However, the profile after the analysis of proteins from the cytoplasmic side of the plasma membrane was different, obtaining the highest percentage after the protein extraction with **buffer 5** (SDS LB) and the lowest with the **buffer 4** (citrate SDS LB) (see Figure 9B).

According to the molecular function of the proteins extracted with the different buffers, small variations were observed in the percentages of proteins related to RNA binding (see Figure 10A), however, more differences were observed for other functions as: ATPase coupled ion transmembrane transporter activity, GTP-dependent protein binding, calcium channel regulator activity, fibrinogen binding and fibroblast growth factor binding (see Figure 10B). As Figure 11A shows, proteins implicated in the biological process of actin filament network formation were extracted with all buffers except for **buffer 6** (RIPA LB). Furthermore, **buffer 4** (citrate-SDS LB) and **buffer 5** (SDS LB) were the only ones that allowed the extraction of proteins that participates in actin-mediated cell contraction, activation of MAPKK activity, adenylate cyclase-activating G-protein coupled receptor signaling pathway and the renal system process (see Figure 11B).

Concerning these results, the deparaffinization method composed of xylene at RT, followed by rehydration with decreasing alcohol percentages and a final hydration step with milli-Q H_2_O allows the complete paraffin removal and, consequently, the protein extraction process seems to be more effective. From the six different protein extraction buffers, SDS-containing **buffer 1** (Tris-SDS LB) and **buffer 5** (SDS LB) promotes the identification of higher number of proteins. Combination of this conditions with a disaggregation step with the TissueLyser seems to be a high effective method for the extraction of high-quality proteins from FFPE kidney tissues specimens.

### Microdissected Glomeruli Study

**Figure 12:**
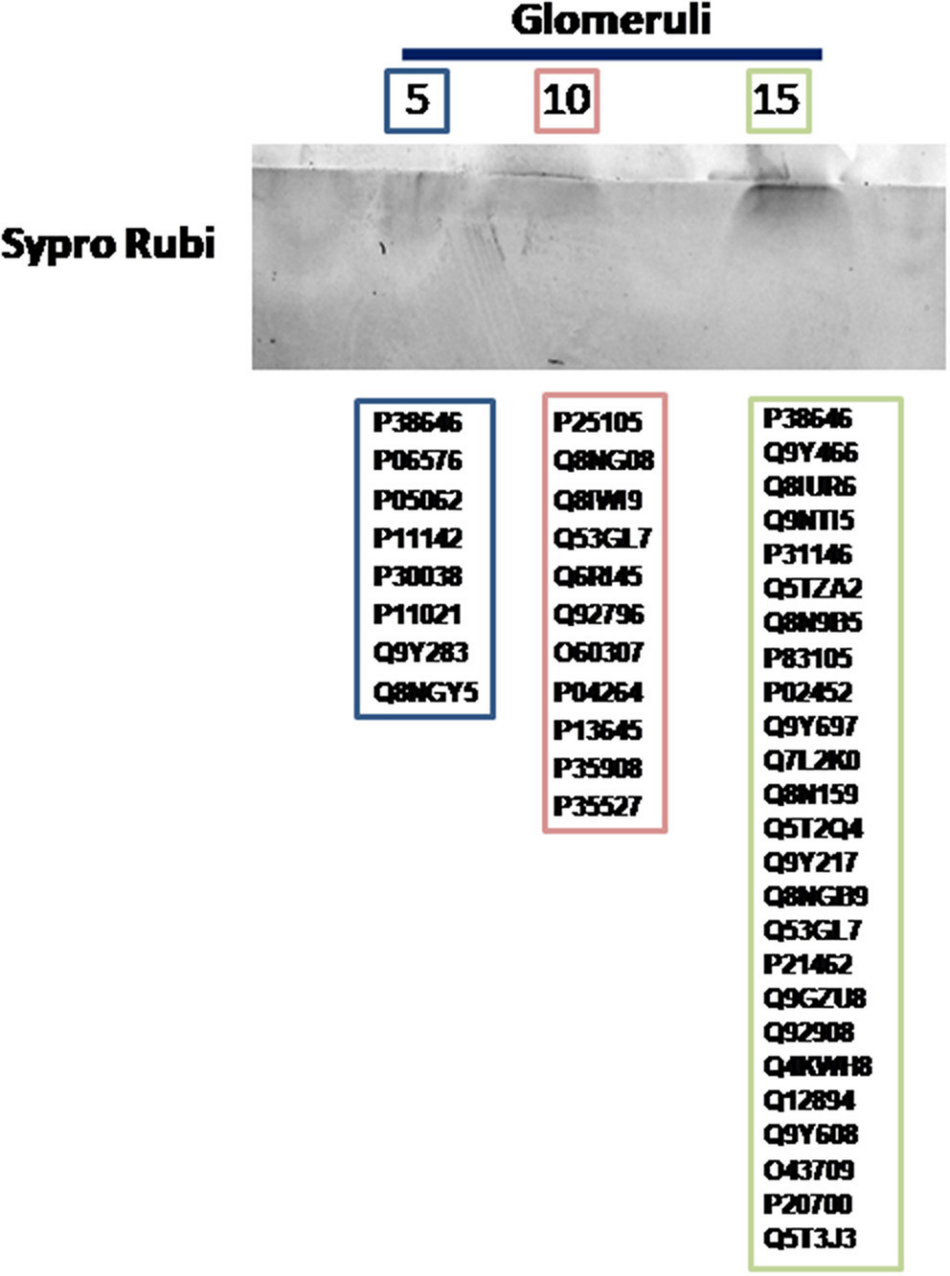
Proteins identified after the application of the optimizated conditions in the present work to the treatment of 5, 10, or 15 microdisected glomeruli.

Previously optimized conditions were applied to the protein extraction from 5, 10, and 15 glomeruli obtained from FFPE renal samples. Importantly, proteins extracted were identified by LC-MS/MS analysis (see Figure 12), showing a total of 8, 10, and 25 proteins, respectively. Further insights are necessary for the application of this methodology in the discovery of biomarkers of different renal disorders, as glomerulonephritis.

## Discussion

The use of FFPE tissue blocks is a standard procedure in the clinical practice as an economical archival choice because tissues can be stored at RT to guarantee long-term stability. These samples representing a wide assortment of biological material to perform different disease process retrospectives. Moreover, these FFPE specimens are often associated with clinical data, including histology reports, treatment, and patient outcomes (12, 28) that may be used to correlate with the findings. However, FFPE samples show some disadvantages because the use of the paraffin-formalin technique induces crosslinks mainly with arginine, lysine, serine, and tyrosine amino acid residues. Furthermore, the formalin-fixation and paraffin-embedded procedure may induce a loss of protein modifications or poor protein retrieval during the protein extraction process, although optical spectroscopic studies indicated that this treatment does not appear to significantly alter the protein's secondary or tertiary structure (12, 44). On the other hand, it is generally accepted that FFPE tissue proteomic data are good enough to be used for biomarker searching, there remains a great variance between protein profiles from FFPE and matched fresh-frozen tissue sections (45).

Based on these observations, some topics must be addressed to assure the accuracy of FFPE tissue proteomics. (1) Which factors affect the efficient and reproducible extraction of proteins from FFPE tissue? Is the paraffin-induced modifications in the protein one of these factors? (2) What is the best protocol to obtain proteins from FFPE tissues that is both reproducible and effective? (3) Is it necessary to establish a universal standard for the evaluation of the quality and quantity of proteins extracted from FFPE tissues, or rather adapt each tissue according to its characteristics? (4) To avoid potential bias due to preferential extraction of proteins by a single protocol, are multiple extraction steps using a range of pH values, buffers, or heating conditions necessary to achieve complete protein coverage? Consequently, to clarify all these questions a protein extraction standardization is necessary (45).

Continuous studies have provided solutions related to the FFPE protein extraction methods to expand the proteomic search. In this way, several papers have been recently published where the authors made a comparison between procedures and buffers to improve the protein extraction of FFPE samples (9, 16, 26, 28, 31, 46).

This work aims to develop a protein extraction protocol from healthy renal FFPE tissue, unlike other studies (47), testing several tissue disaggregation methods, deparaffinization procedures, and protein extraction buffers for proteomics analysis.

One of the most critical steps in the proteomic field is the quantitative recovery of proteins from samples before their subsequent proteomic analysis (45). While protein recovery can be complicated in some fresh or frozen tissues, in FFPE the dilemma is especially challenging.

A correct tissue homogenization is fundamental to guarantee the total buffer penetration in the tissue sample; thus, it is an important procedure that needs standardization in the management of FFPE samples. In this way, mechanical disaggregation showed to be useful for the treatment of different tissue types (48, 49).

In the present study, the **protocol 3** offered the highest quality and quantity of proteins, where FFPE tissue samples were deparaffinated with xylene (at 65°C) or mineral oil (at 95°C) in combination with a mechanic TissueLyser disaggregation. Probably, this procedure promotes the elimination of the remaining paraffin of the sample. Although several papers reported the use of higher temperature combined with moderate pressure (15 psi) (50), or the use of a modified Laemmli protocol (19, 20) to obtain a good tissue homogenization, in the present work the use of pressure (syringe extraction) does not show good results.

In the deparaffination step, the complete solubilization of formalin promotes the protein extraction for the subsequent proteomic analysis (45). However, two main problems must be addressed: the insolubility of paraffin in water and the chemical modifications of proteins. Although different solvents were used for the paraffin removal, generally, the solubilization is achieved using apolar solvents and the rehydration is carried out with alcohols at different concentrations (7). As we previously described, xylene is the most widely used apolar solvent, but some authors also used mineral oil as a non-toxic (7, 31) and effective reagent or, in some cases, even heptane (51). In the present work, three deparaffinization methods were tested (see section **Deparaffinization Methods**). While the poor results obtained with mineral oil al 95°C could be due to an incomplete deparaffinization with this solvent that impeded the protein extraction buffer penetration, the bad results obtained with xylene at 65°C were probably due to the degradation of proteins at this temperature. These drawbacks were avoided after using xylene at RT following a method previously reported method (42, 52).

As it as mentioned above, the protein extraction efficiency could be modulated by some factors such as temperature, incubation time, extraction buffer composition, application of an ultrasonic method or other methods for tissue disaggregation (7, 14). In this way, it was previously described that heating the tissue in the presence of the extraction buffer promotes the protein extraction though the elimination of intra- and intermolecular cross-links (42). Furthermore, the protein extraction efficiency will be higher with the use of an aggressive disaggregation method (as TissueLyser) that promotes the reaction between the proteins and the extraction buffer.

Importantly, the method developed in the present work was also effective for the protein extraction in 1.5 mg of FFPE tissue samples, an amount lower than those used in previous works. Comparatively, R. W. Sprung et al. (23) developed an SRM study where they used sections of 30 μm (over 35 mg), 10 times more than that employed in the present study. Other studies used three serial sections of 10 μm thickness and xylene and xylol as deparaffination agent or sections of 10 μm and heptane as deparaffination agent (51). Moreover, we can obtain protein we perform a previously microdissection and only using 5 glomeruli in our analysis (Figure 12).

As we mentioned above, the incomplete solubilization of proteins and reversal of formaldehyde adducts and crosslinks from FFPE tissues can reduce accurate protein analysis and can fail to identify constituent proteins in the extracts. In this way, shotgun LC-MS/MS is established as one of the most powerful and widespread approaches for the proteomic characterization of complex samples such as FFPE tissue extracts. After the optimization of the deparaffinization and the disaggregation steps, a mass spectrometry analysis was developed to select the best conditions after compare the type and the number of proteins identified with each protein extraction buffers of different composition. It is important to mention that the number of proteins identified in the present work (see Table 3) is similar or even higher than that reached by other authors. For example, Sprung et al. (23) identified a total of 1982 proteins in FFPE tissue samples but using an amount of protein 10 times higher. Nirmalan et al. (20) obtained a similar number of identified proteins but using xylene for the deparaffinization step and Rapigest for the direct protein digestion.

Thus, the efficiency of six buffers with various pH values (ranging from pH 6.8 to 8) and different composition were tested to compare by mass spectrometry the quantity and quality of proteins extracted from FFPE tissues. In relation with the pH, some authors suggest that an extremely high pH can induce poor protein recovery, whilst a low pH can produce breaks by the acidification of the aspartic acid, producing a lesser protein yield (24). However, other authors obtained better results with pH 8 or greater, because it facilitates the breaking of methylene bridges, thus enabling protein release (7, 26). Araujo et al. considered pHs 7.4 and 9 as best for their experiment design (42). In the present work, the efficiency of the extraction (higher number of identified proteins) was better at pH 8 with the **buffer 1** (Tris-SDS LB) identifying 979 proteins, pH 7.5 with **buffer 4** (citrate-SDS LB) identifying 921 proteins, and at pH 6.8 with **buffer 3** (Tris-SDS-DTT-Glycerol LB) and **buffer 5** (SDS LB), identifying 930 and 1,086 proteins.

Another key factor are detergents presented in the protein extraction buffer that promotes the solubilization of proteins presented in FFPE tissues samples (42). In the present work, buffers contain detergents as Triton X-100 (**buffer 6**), CHAPS (**buffer 2**), or the anionic detergent SDS (**buffer 1**, **buffer 3**, **buffer 4**, **buffer 5** and **buffer 6**). As can be seen in Figure 3B and Table 3, the use of SDS-containing buffers in a percentage between 2–5% promotes proteins extraction, something reported previously by different authors (31, 32, 46). Furthermore, it was also described that a higher % of SDS hampers the solubilization of hydrophilic proteins (52). SDS contributed to the protein extraction thought the denaturalization of native proteins into their polypeptides, which could be enhanced after the incubation at 100°C. However, the combination of SDS with Triton X-100 (a weak protein-denaturing agent) in **buffer 6**, does not enhance protein extraction (52).

On the other hand, some authors suggest that the combination of detergents, denaturant agents and reductants plays an important role in the protein extraction since this combination could induce protein unfolding increasing the protein accessibility from formalin-fixed samples (26). In the present work, this statement was confirmed because the combination of the detergent SDS and the reductant agent DTT in **buffer 1** (Tris-SDS LB) and **buffer 3** (Tris-SDS-DTT-Glycerol LB) contributed to the protein extraction, identifying 979 and 930 proteins, respectively.

Examples of the use of SDS-containing buffers to extract proteins from FFPE kidney tissue samples were also reported. For example, Craven et al. (15) obtained over 2000 proteins from 5 cm^2^ renal cell carcinoma FFPE samples using a 4% SDS-containing buffer. Ostasiewicz et al. (14) showed that a buffer containing Tris-hydrochloric acid, DTT, and SDS extracted 2,938 proteins from 10 FFPE slices of 10 μm thickness although, in this case, the analysis was performed with liver samples.

Despite the effectiveness of SDS-containing buffer, its incompatibility with MS and with the previous enzymatic digestion makes it necessary for an SDS removal. In the present work, proteins were concentrated in an SDS-PAGE gel to the subsequent “in-gel” digestion. From the 468 proteins commonly identified after the use of all SDS-containing extraction buffers, more than 80% were previously found to be expressed in kidney tissue. Some of these proteins are uromodulin, transporters as ADP/ATP translocase 2, mitochondrial 2-oxoglutarate/malate carrier protein, ezrin-radixin-moesin-binding phosphoprotein 50 and band 3 anion transport protein, among others, mainly related to the filter function of the kidney. Other proteins such as clusterin, serum amyloid P-component, complements, collagenases, apolipoprotein E, serpin, transthyretin, fatty acid-binding protein, immunoglobulins, keratins, ferritin, light and heavy chain, heparan sulfate proteoglycan, vinculin, transgelin, glyceraldehyde-3-phosphate dehydrogenase, pyruvate kinase orS100 were described previously by other authors in kidney samples (25, 26, 42, 43).

From the 468 common proteins, 3.7% were involved in the extracellular matrix organization and 4.1% in the innate immune response. These results agree with kidney biology because their function relies strongly on the adequate regulation of the extracellular matrix. Therefore, deregulation in the extracellular matrix in the kidney is involved in many glomerular diseases and is a final common pathway of glomerular injury (53). Moreover, the immune system is intricately linked to these organs. On the one hand, in a healthy state, kidneys contribute to immune homeostasis, and on the other hand components of the immune system mediate many acute forms of renal disease playing a central role in the progression of chronic kidney disease. Additionally, it is important to mention that a dysregulated immune system can have direct or indirect renal effects (54, 55). In relation with their molecular function, some of these common proteins are important in kidney physiology and their architecture and function are maintained and regulated by actin and several actin-binding proteins. Mutations at these levels can produce glomerular diseases (56).

Although the problem of the SDS incompatibility in mass spectrometry was solved by SDS-PAGE gel and “in-gel” digestion, the urea-containing **buffer 2** (urea LB) was also tested. This buffer used urea as a substitute for a chaotropic reagent (26, 52). However, this approach has certain limitations because lytic strength is poor and leaves some insoluble proteins behind, such as membrane proteins; it makes a less efficient protein digestion and peptide identification.

Functional analysis revealed that proteins with different molecular and biological function could be extracted from the same FFPE tissue specimen, depending on the buffer selected: SDS-containing buffers (**buffer 1**, **buffer 3**, **buffer 4**, **buffer 5**, **buffer 6**) and the urea-containing buffer (**buffer 2**).

In summary, the present work demonstrates that a successful identification of proteins extracted from FFPE kidney tissue samples can be performed by LC-MS/MS taking into account the following considerations: (i) xylene at RT can eliminate the formalin fixation efficiently from the FFPE kidney tissue specimens, (ii) both, high-temperature (at least 20 min at 100°C), the presence of strong detergents (SDS or analog) and reducing agents (e.g., DTT) in the extraction buffer, seem to be factors that contribute to obtain good extraction yields; (iii) buffer pH values should be in the range 7–9 (only slight variations in the protein extraction yield are observed within that range); (iv) tissue homogenization/disaggregation using a TissueLyser and sonication treatment can improve the extraction efficiency. Regarding the minimal amount of tissue sample necessary to obtain good quality protein, the present work demonstrated that can obtain protein from 1.5 mg of FFPE tissue. This method allows obtaining protein using much less amount of tissue than the method proposes by other authors. Importantly, the methodology proposed in this work was effective for the extraction of proteins from only 5 glomeruli, open a new way in the search of novel biomarkers of different renal diseases.

## Data Availability Statement

The datasets generated for this study can be found in online repositories. The names of the repository/repositories and accession number(s) can be found below: PRIDE repository; Project Name: Protein extraction method improvement for FFPE kidney tissue samples; Project accession: PXD023891.

## Ethics Statement

The studies involving human participants were reviewed and approved by D. Alfonso Casas Losada (Presidente). Médico especialista en Psiquiatría D. Santiago Pérez Cachafeiro (Vicepresidente). Médico Especialista en Medicina Familiar y Comunitaria. Dª. Asunción Verdejo González (Secretaria). Médica Especialista en Farmacología Clínica. D. Víctor del Campo Pérez (Secretario Suplente). Médico Especialista en Medicina Preventiva y Salud Pública. Dª. Iria Aparicio Rodríguez. Médica especialista en Obstetricia y Ginecología. Dª. Aurelia Constenla Castro. Diplomada Universitaria de Enfermería. Dª. M.ª Dolores Martínez Pereira. Licenciada en Derecho. D. Jorge Luis Arias Otero. Licenciado en Físicas. D. Adolfo Paradela Carreiro. Farmacéutico de Atención Especializada. Dª. María Eva Pérez López. Médica Especialista en Oncología Médica. Dª. Maria de las Mercedes Guerra García. Farmacéutica de Atención Primaria. Dª. Cristina Torreira Banzas. Médica Especialista en Análisis Clínicos. Dª. Miriam Vázquez Campo. Diplomada Universitaria de Enfermería. The patients/participants provided their written informed consent to participate in this study.

## Author Contributions

SB, JB, CN, and JC-T: conceptualization. MG-V, MC-V, AS-F, RA, and AB: methodology. MC-V, MG-V, SB, and CN: software and investigation. MG-V, MC-V, JB, SB, JC-T, AO-G, MG-G, and CN: formal analysis. MC-V, MG-V, SB, JB, and CN: resources. SB, JB, and CN: writing-original draft preparation. SB, JB, JC-T, and CN: writing-review and editing. SB and CN: visualization. JB, SB, and CN: supervision. SB: project administration. All authors have read and agreed to the published version of the manuscript.

## Conflict of Interest

The authors declare that the research was conducted in the absence of any commercial or financial relationships that could be construed as a potential conflict of interest.

## Supplementary Material

Supplementary Table 1 Identified proteins by LC-MS/MS using the six different extraction buffers. Data of experimental replicates (*x3*) are shown.

Supplementary Table 2 Identified proteins in by LC-MS/MS using the SDS-containing buffers for the extraction of proteins from FFPE kidney tissue samples: **buffer 1** (Tris-SDS LB), **buffer 3** (Tris-SDS-DTT-Glycerol LB), **buffer 4** (citrate-SDS LB), **buffer 5** (SDS-LB), and **buffer 6** (RIPA LB). Proteins are distributed in color corresponding to the Venn diagram showed in Figure 5.

Supplementary Table 3 List of the 468 common proteins identified after the extraction with the five SDS-containing buffers for their functional classification according to their biological process, molecular function and cellular component, using the FunRich program.

Supplementary Table 4 List of proteins and functional classification of the 896 and 21 unique proteins identified after the extraction with the five SDS-containing buffers [**buffer 1** (Tris-SDS LB), **buffer 3** (Tris-SDS-DTT-Glycerol LB), **buffer 4** (citrate-SDS LB), **buffer 5** (SDS-LB), and **buffer 6** (RIPA LB)] and the urea-containing buffer [**buffer 2** (urea LB)] (red bars), respectively, according to their cellular component, molecular function and (B) biological process, using the FunRich program.

Supplementary Table 5 Functional classification of all proteins identified after the extraction with the six different buffers [**buffer 1** (Tris-SDS LB), *n* = 979 proteins; **buffer 2** (urea LB), *n* = 569 proteins; **buffer 3** (Tris-SDS-DTT-Glycerol LB), *n* = 930 proteins; **buffer 4** (citrate-SDS LB), *n* = 921 proteins; **buffer 5** (SDS-LB), *n* = 1086 proteins; **buffer 6** (RIPA LB), *n* = 540 proteins], according to their biological process, molecular function and cellular components, using the FunRich program.

## References

[1] Booth FW Gordon SE Carlson CJ Hamilton MT. Waging war on modern chronic diseases: primary prevention through exercise biology. J Appl Physiol. (2000) 88:774–87. 10.1152/jappl.2000.88.2.774 10658050

[2] Ma C-M Lu N Wang R Liu X-L Lu Q Yin F-Z. Three novel obese indicators perform better in monitoring management of metabolic syndrome in type 2 diabetes. Sci Rep. (2017) 7:9843. 10.1038/s41598-017-10446-3 28852155 PMC5574991

[3] Wang Z Yuan D Duan Y Li S Hou S. Key factors involved in obesity development. Eat Weight Disord. (2018) 23:267–74. 10.1007/s40519-017-0428-3 28840575

[4] Jain KK. Role of Proteomics in the Development of Personalized Medicine. Adv Protein Chem Struct Biol. (2016) 102:41–52. 10.1016/bs.apcsb.2015.09.002 26827601

[5] Grillo F Bruzzone M Pigozzi S Prosapio S Migliora P Fiocca R. Immunohistochemistry on old archival paraffin blocks: i s there an expiry date? J Clin Pathol. (2017) 70:988–93. 10.1136/jclinpath-2017-204387 28596153

[6] Hood BL Darfler MM Guiel TG Furusato B Lucas DA Ringeisen BR. Proteomic analysis of formalin-fixed prostate cancer tissue. Mol Cell Proteomics. (2005) 4:1741–53. 10.1074/mcp.M500102-MCP200 16091476

[7] Giusti L Lucacchini A. Proteomic studies of formalin - fixed paraffin - embedded tissues. Expert Rev Proteomics. (2013) 10:165–77. 10.1586/epr.13.3 23573783

[8] Weißer J Lai ZW Bronsert P Kuehs M Drendel V Timme S. Quantitative proteomic analysis of formalin–fixed, paraffin–embedded clear cell renal cell carcinoma tissue using stable isotopic dimethylation of primary amines. BMC Genomics. (2015) 16:559. 10.1186/s12864-015-1768-x 26220445 PMC4518706

[9] Coscia F Doll S Bech JM Schweizer L Mund A Lengyel E. A streamlined mass spectrometry-based proteomics workflow for large scale FFPE tissue analysis. J Pathol. (2020) 251:100–12. 10.1002/path.5420 32154592

[10] García-Vence M Chantada-Vázquez MDP Cameselle-Teijeiro JM Bravo SB Núñez C. A novel nanoproteomic approach for the identification of molecular targets associated with thyroid tumors. Nanomaterials (Basel). (2020) 10:2370. 10.3390/nano10122370 33260544 PMC7761166

[11] Gustafsson OJR Arentz G Hoffmann P. Proteomic developments in the analysis of formalin-fixed tissue. Biochim Biophys Acta. (2015) 1854:559–80. 10.1016/j.bbapap.2014.10.003 25315853

[12] O'Rourke MB Padula MP O'Rourke MB Padula MP. Analysis of formalin-fixed, paraffin-embedded (FFPE) tissue via proteomic techniques and misconceptions of antigen retrieval. BioTechniques. (2016) 60:229–38. 10.2144/000114414 27177815

[13] Palmer-toy DE Krastins B Sarracino DA Nadol JB Merchant SN. Efficient method for the proteomic analysis of fixed and embedded tissues. J Proteome Res. (2005) 2404–11. 10.1021/pr050208p 16335994

[14] Ostasiewicz P Zielinska DF Mann M Wisniewski JR. Proteome, phosphoproteome, and N-glycoproteome are quantitatively preserved in formalin-fixed paraffin-embedded tissue and analyzable by high-resolution mass spectrometry. J Proteome Res. (2010) 9:3688–700. 10.1021/pr100234w 20469934

[15] Craven RA Cairns DA Zougman A Harnden P Selby PJ Banks RE. Proteomic analysis of formalin-fixed paraffin-embedded renal tissue samples by label-free MS: assessment of overall technical variability and the impact of block age. Proteomics Clin Appl. (2013) 7:273–82. 10.1002/prca.201200065 23027403

[16] Kuras M Woldmar N Kim Y Hefner M Malm J Moldvay J. Proteomic workflows for high-quality quantitative proteome and post-translational modification analysis of clinically relevant samples from formalin-fixed paraffin-embedded archives. J Proteome Res. (2021) 20:1027–39. 10.1021/acs.jproteome.0c00850 33301673

[17] Shi S-R Shi Y Taylor CR. Antigen retrieval immunohistochemistry: review and future prospects in research and diagnosis over two decades. J Histochem Cytochem. (2011) 59:13–32. 10.1369/jhc.2010.957191 21339172 PMC3201121

[18] Shi SR Key ME Kalra KL. Antigen retrieval in formalin-fixed, paraffin-embedded tissues: an enhancement method for immunohistochemical staining based on microwave oven heating of tissue sections. J Histochem Cytochem. (1991) 39:741–8. 10.1177/39.6.1709656 1709656

[19] Nirmalan NJ Harnden P Selby PJ Banks RE. Development and validation of a novel protein extraction methodology for quantitation of protein expression in formalin-fixed paraffin-embedded tissues using western blotting. J Pathol. (2009) 217:497–506. 10.1002/path.2504 19156775

[20] Nirmalan NJ Hughes C Peng J McKenna T Langridge J Cairns DA. Initial development and validation of a novel extraction method for quantitative mining of the formalin-fixed, paraffin-embedded tissue proteome for biomarker investigations. J Proteome Res. (2011) 10:896–906. 10.1021/pr100812d 21117664 PMC3033703

[21] Perroud B Lee J Valkova N Dhirapong A Lin P-Y Fiehn O. Pathway analysis of kidney cancer using proteomics and metabolic profiling. Mol Cancer. (2006) 5:64. 10.1186/1476-4598-5-64 17123452 PMC1665458

[22] Perroud B Ishimaru T Borowsky AD Weiss RH. Grade-dependent proteomics characterization of kidney cancer. Mol Cell Proteomics. (2009) 8:971–85. 10.1074/mcp.M800252-MCP200 19164279 PMC2689781

[23] Sprung RW Martinez MA Carpenter KL Ham A-JL Washington MK Arteaga CL. Precision of multiple reaction monitoring mass spectrometry analysis of formalin-fixed, paraffin-embedded tissue. J Proteome Res. (2012) 11:3498–505. 10.1021/pr300130t 22530795 PMC3368395

[24] Guo H Liu W Ju Z Tamboli P Jonasch E Mills GB. An efficient procedure for protein extraction from formalin-fixed, Paraffin-embedded tissues for reverse phase protein arrays. Proteome Sci. (2012) 10:56. 10.1186/1477-5956-10-56 23006314 PMC3561137

[25] Finne K Vethe H Skogstrand T Leh S Dahl TD Tenstad O. Proteomic analysis of formalin-fixed paraffin-embedded' glomeruli suggest depletion of glomerular filtration barrier proteins in two-kidney, one-clip hypertensive rats. Nephrol Dial Transplant. (2014) 29:2217–27. 10.1093/ndt/gfu268 25129444 PMC4240179

[26] Shen K Sun J Cao X Zhou D Li J. Comparison of different buffers for protein extraction from formalin-fixed and paraffin-embedded tissue specimens. PLoS ONE. (2015) 10:e0142650. 10.1371/journal.pone.0142650 26580073 PMC4651363

[27] Srivastava S Merchant M Rai A Rai SN. Standardizing proteomics workflow for liquid chromatography-mass spectrometry: technical and statistical considerations. J Proteomics Bioinform. (2019) 12:48–55. 10.35248/0974-276x.19.12.496 32148359 PMC7059694

[28] Giusti L Angeloni C Lucacchini A. Update on proteomic studies of formalin-fixed paraffin-embedded tissues. Expert Rev Proteomics. (2019) 16:513–20. 10.1080/14789450.2019.1615452 31094245

[29] Bayer M Angenendt L Schliemann C Hartmann W König S. Are formalin-fixed and paraffin-embedded tissues fit for proteomic analysis? J Mass Spectrometry. (2019) 55:e4347. 10.1002/jms.4347 30828905

[30] Ostasiewicz PP Zielinska DF Mann M Wiśniewski JR Wisniewski JR Wisniewski JR. Proteome, phosphoproteome, and N-glycoproteome are quantitatively preserved in formalin-fixed paraffin-embedded tissue and analyzable by high-resolution mass spectrometry. J Proteome Res. (2010) 9:3688–700. 20469934 10.1021/pr100234w

[31] Rodríguez-Rigueiro T Valladares-Ayerbes M Haz-Conde M Blanco M Aparicio G Fernández-Puente P. A novel procedure for protein extraction from formalin-fixed paraffin-embedded tissues. Proteomics. (2011) 11:2555–9. 10.1002/pmic.201000809 21591256

[32] Vincenti DC Murray GI. The proteomics of formalin-fixed wax-embedded tissue. Clin Biochem. (2013) 46:546–51. 10.1586/14789450.2013.820531 23063984

[33] Bonzon-Kulichenko E Pérez-Hernández D Núñez E Martínez-Acedo P Navarro P Trevisan-Herraz M. A robust method for quantitative high-throughput analysis of proteomes by 18O labeling. Mol Cell Proteomics. (2011) 10:M110.003335. 10.1074/mcp.M110.003335 20807836 PMC3013457

[34] Perez-Hernandez D Gutiérrez-Vázquez C Jorge I López-Martín S Ursa A Sánchez-Madrid F. The intracellular interactome of tetraspanin-enriched microdomains reveals their function as sorting machineries toward exosomes. J Biol Chem. (2013) 288:11649–61. 10.1074/jbc.M112.445304 23463506 PMC3636856

[35] Shevchenko A Wilm M Vorm O Mann M. Mass spectrometric sequencing of proteins silver-stained polyacrylamide gels. Anal Chem. (1996) 68:850–8. 10.1021/ac950914h 8779443

[36] Paradela-Dobarro B Bravo SBSB Rozados-Luís A González-Peteiro M Varela-Román A González-Juanatey JR. Inflammatory effects of *in vivo* glycated albumin from cardiovascular patients. Biomed Pharmacother. (2019) 113:108763. 10.1016/j.biopha.2019.108763 30875658

[37] del Pilar Chantada-Vázquez M López AC Vence MG Vázquez-Estévez S Acea-Nebril B Calatayud DG. Proteomic investigation on bio-corona of Au, Ag and Fe nanoparticles for the discovery of triple negative breast cancer serum protein biomarkers. J Proteomics. (2020) 212:103581. 10.1016/j.jprot.2019.103581 31731051

[38] Hermida-Nogueira L Barrachina MN Izquierdo I García-Vence M Lacerenza S Bravo S. Proteomic analysis of extracellular vesicles derived from platelet concentrates treated with Mirasol® identifies biomarkers of platelet storage lesion. J Proteomics. (2020) 210:103529. 10.1016/j.jprot.2019.103529 31605789

[39] Tamara C Nerea L-B Belén BS Aurelio S Iván C Fernando S. Vesicles shed by pathological murine adipocytes spread pathology: characterization and functional role of insulin resistant/hypertrophied adiposomes. Int J Mol Sci. (2020) 21:2252. 10.3390/ijms21062252 32214011 PMC7139903

[40] Varela-Rodríguez BM Juiz-Valiña P Varela L Outeiriño-Blanco E Bravo SB García-Brao MJ. Beneficial effects of bariatric surgery-induced by weight loss on the proteome of abdominal subcutaneous adipose tissue. J Clin Med. (2020) 9:213. 10.3390/jcm9010213 31941045 PMC7019912

[41] Teodora F Paunas I Finne K Leh S Al-Hadi Osman T Marti. Characterization of glomerular extracellular matrix in IgA nephropathy by proteomic analysis of laser-captured microdissected glomeruli. BMC Nephrol. (2019) 20:410. 10.1186/s12882-019-1598-1 31726998 PMC6854890

[42] Araújo JE Oliveira E Otero-Glez A Santos Nores J Igrejas G Lodeiro C. A comprehensive factorial design study of variables affecting protein extraction from formalin-fixed kidney tissue samples. Talanta. (2014) 119:90–97. 10.1016/j.talanta.2013.10.019 24401389

[43] Blutke A. Opening a treasure chest: glomerular proteome analyses of formalin-fixed paraffin-embedded kidney tissue in the investigation of diabetic nephropathy. Nephrol Dial Transplant. (2012) 27:1695–8. 10.1093/ndt/gfs082 22547746

[44] Rait VK O'Leary TJ Mason JT. Modeling formalin fixation and antigen retrieval with bovine pancreatic ribonuclease A: I—Structural and functional alterations. Lab Invest. (2004) 84:292–9. 10.1038/labinvest.3700045 14968117 PMC1747597

[45] Shi S-R Taylor CR Fowler CB Mason JT. Complete solubilization of formalin-fixed, paraffin-embedded tissue may improve proteomic studies. Proteomics Clin Appl. (2013) 7:264–72. 10.1002/prca.201200031 23339100 PMC3779364

[46] Shi S-R Liu C Balgley BM Lee Taylor CCR. Protein extraction from formalin-fixed, paraffin-embedded tissue sections: quality evaluation by mass spectrometry. J Histochem Cytochem. (2006) 54:739–43. 10.1369/jhc.5B6851.2006 16399996

[47] Wolff C Schott C Porschewski P Reischauer B Becker K.-F. Successful protein extraction from over-fixed and long-term stored formalin-fixed tissues. PLoS ONE. (2011) 6:e16353. 10.1371/journal.pone.0016353 21305021 PMC3031559

[48] Lee C. Protein extraction from mammalian tissues. Methods Mol Biol. (2007) 362:385–9. 10.1007/978-1-59745-257-1_29 17417026

[49] Goldberg S. Mechanical/physical methods of cell distribution and tissue homogenization. Methods Mol Biol. (2015) 1295:1–20. 10.1007/978-1-4939-2550-6_1 25820709

[50] Chung JY Lee SJ Kris Y Braunschweig T Traicoff JL Hewitt SM. A well-based reverse-phase protein array applicable to extracts from formalin-fixed paraffin-embedded tissue. Proteomics Clin Appl. (2008) 2:1539–47. 10.1002/prca.200800005 21136801 PMC3777740

[51] Hammer E Ernst FD Thiele A Karanam NK Kujath C Evert M. Kidney protein profiling of Wilms' tumor patients by analysis of formalin-fixed paraffin-embedded tissue samples. Clin Chim Acta. (2014) 433:235–41. 10.1016/j.cca.2014.03.020 24680863

[52] Guo T Wang W Rudnick PA Song T Li J Zhuang Z. Proteome analysis of microdissected formalin-fixed and paraffin-embedded tissue specimens. J Histochem Cytochem. (2007) 55, 763–72. 10.1369/jhc.7A7177.2007 17409379

[53] Hobeika L Barati MT Caster DJ McLeish KR Merchant ML. Characterization of glomerular extracellular matrix by proteomic analysis of laser-captured microdissected glomeruli. Kidney Int. (2017) 91:501–11. 10.1016/j.kint.2016.09.044 27988214 PMC5237413

[54] Wang Y-H Zhang Y-G. Kidney and innate immunity. Immunol Lett. (2017) 183:73–8. 10.1016/j.imlet.2017.01.011 28143791

[55] Tecklenborg J Clayton D Siebert S Coley SM. The role of the immune system in kidney disease. Clin Exp Immunol. (2018) 192:142–50. 10.1111/cei.13119 29453850 PMC5904695

[56] Ludwig-Peitsch WK. Drebrin in renal glomeruli. Adv Exp Med Biol. (2017) 1006:337–45. 10.1007/978-4-431-56550-5_20 28865030

